# 16 weeks of moderate intensity resistance exercise improves strength but is insufficient to alter brain structure in Gulf War Veterans with chronic musculoskeletal pain: a randomized controlled trial

**DOI:** 10.3389/fnins.2025.1488397

**Published:** 2025-04-16

**Authors:** Stephanie M. Van Riper, Jacob V. Ninneman, Aaron J. Stegner, Brady A. Riedner, Laura D. Ellingson, Ryan J. Dougherty, Patrick J. O’Connor, Gunnar A. Roberge, Andrew L. Alexander, Doug C. Dean, Jill N. Barnes, Dane B. Cook

**Affiliations:** ^1^Center for Healthy Minds, University of Wisconsin, Madison, WI, United States; ^2^Research Service, William S. Middleton Memorial Veterans Hospital, Madison, WI, United States; ^3^Department of Kinesiology, University of Wisconsin, Madison, WI, United States; ^4^Division of Health and Exercise Science, Western Oregon University, Monmouth, OR, United States; ^5^Department of Kinesiology and Health, Rutgers University, New Brunswick, NJ, United States; ^6^Department of Kinesiology, University of Georgia, Athens, GA, United States; ^7^Waisman Center, Brain Imaging Core, University of Wisconsin, Madison, WI, United States; ^8^Department of Medical Physics, University of Wisconsin, Madison, WI, United States; ^9^Department of Psychiatry, University of Wisconsin, Madison, WI, United States; ^10^Department of Pediatrics, University of Wisconsin, Madison, WI, United States

**Keywords:** exercise, clinical trial, chronic pain, brain, Gulf War illness

## Abstract

**Introduction:**

Chronic widespread musculoskeletal pain (CMP) is a primary condition of Veterans who were deployed to the Persian Gulf War. The mechanisms that underlie CMP in these Veterans are unknown and few efficacious treatment options exist. This study tested the effects of 16 weeks of resistance exercise training (RET) on gray matter (GM) volume and white matter (WM) microstructure in Gulf War Veterans (GWVs) with CMP compared to GWV waitlist controls (WLC).

**Methods:**

Fifty-four GWVs were randomly assigned to 16 weeks of RET (*n* = 28) or WLC (*n* = 26). Training involved 10 resistance exercises to involve the whole body, was supervised and individually tailored, and progressed slowly to avoid symptom exacerbation. Outcomes assessed at baseline, 6, 11 and 17 weeks and 6- and 12-months post-intervention included GM volume (voxel-based morphometry), WM microstructure (diffusion tensor imaging), pain [short form McGill Pain Questionnaire (SF-MPQ) and 0–100 visual analog scale (VAS)], fatigue (0–100 VAS), and mood (Profile of Mood States). Muscular strength was assessed at baseline, 8 and 16 weeks, and training volume was tracked throughout the 16-week intervention. Primary analyses used linear mixed effects models with Group, Time, and the Group*Time interaction as fixed factors and subject and slope as random factors to test the differential effects of RET and WLC on brain structure and symptoms. All neuroimaging analyses used the False Discovery Rate to correct for multiple comparisons at an alpha of 0.05.

**Results:**

Strength increased significantly across the trial for the RET group (*p* < 0.001). There were significant Group*Time interaction effects for pain ratings (SF-MPQ total; *p* < 0.01) and the Profile of Mood States total mood disturbance score (*p* < 0.01). There were no Group or Group*Time effects for GM volume or WM microstructure. There were no significant associations between strength, symptoms, and brain structure (*p* > 0.05).

**Conclusion:**

Sixteen weeks of low-to-moderate intensity RET (i) improved musculoskeletal strength and (ii) did not exacerbate symptoms, but (iii) was insufficient to alter brain structure in GWVs with CMP.

## Introduction

Despite decades of research, deployed Gulf War Veterans (GWVs) continue to experience chronic and multisymptom illnesses (Institute of Medicine, 2014). Epidemiology research has consistently illustrated the diverse array of symptoms reported by GWVs – principally pain, fatigue, problems with concentration and memory (i.e., brain fog), and disturbed sleep (Institute of Medicine, 2014). These symptoms occur at rates disproportional to Veterans of the same era who were not deployed to the Persian Gulf region (Blanchard et al., 2006; Kang et al., 2000; Kang et al., 2009), and are significantly and positively associated with functional impairment and health care utilization (Blanchard et al., 2006; Kang et al., 2000; Kang et al., 2009). Among the diverse symptoms, pain is one of the most prevalent—reported by ~30% of GWVs (Institute of Medicine, 2014). Moreover, Veterans deployed to Operations Enduring Freedom (2001) and Iraqi Freedom (2003) report experiencing pain symptoms at rates equal to or exceeding those of GWVs (Gironda et al., 2006; Haskell et al., 2009; Helmer et al., 2009; Lewis et al., 2012). Despite the substantial prevalence of pain in GWVs, few studies have focused on this specific chronic condition, and no efficacious treatments are widely available.

The pathophysiology of chronic musculoskeletal pain (CMP) in GWVs is likewise understudied and poorly understood. Brain mechanisms of CMP, often discussed as central nervous system dysregulation of sensory processing and deficits in the endogenous descending pain inhibitory systems have been explored to some extent (Ossipov et al., 2014). Pain psychophysics and functional brain imaging studies have shown that GWVs with CMP exhibit augmented responses to nociceptive stimuli (Cook et al., 2010; Gopinath et al., 2012; Lindheimer et al., 2019; Dunphy et al., 2003) and that pain interferes with functional brain responses during cognitive processing (Lindheimer et al., 2021). Structural brain imaging studies have shown that GWVs with CMP have smaller gray matter (GM) volumes in brain regions involved in pain regulation and larger GM volumes in regions involved in pain sensitivity and emotion regulation (Ninneman et al., 2022); and altered white matter (WM) microstructure along several pain relevant WM tracts (Van Riper et al., 2017). Importantly, these studies demonstrated GM volumes and disruption of WM microstructure were significantly associated with chronic pain symptoms (Ninneman et al., 2022; Van Riper et al., 2017). These findings, in consideration with chronic pain research in civilians, suggest that CMP in GWVs involves disruption of brain structure and function (Barroso et al., 2021). Treatments capable of mitigating disruptions in brain structure and function may be beneficial for these patients. To this end, exercise training has emerged as a potential therapy capable of stimulating neuroplasticity primarily in animal models, and with some limited evidence in humans (van Praag et al., 1999; Colcombe et al., 2006; Erickson et al., 2011).

Although there are no consistently efficacious treatments for GWVs with chronic pain, chronic exercise training [including resistance exercise training (RET)] is considered a critical element of the multidisciplinary treatment approach for a variety of chronic pain conditions such as fibromyalgia (FM), chronic low back pain, osteoarthritis, and rheumatoid arthritis; and a substantial body of literature has demonstrated its beneficial effects on pain symptoms, physical function, and a host of other clinically relevant outcomes (Clauw, 2014; Häuser et al., 2010; Juhl et al., 2014; Koes et al., 2010; Macfarlane et al., 2017; Oesch et al., 2010; Pedersen and Saltin, 2015; Rainville et al., 2004; Ninneman et al., 2024). Exercise training data in GWVs, however, is sparse. A systematic review of treatment interventions (Nugent et al., 2021) identified a single randomized controlled trial of aerobic exercise training in Gulf War illness (Donta and Clauw, 2003), reporting modest improvements in fatigue and mental health functioning. We previously reported that a 16–week RET program improved strength and disease perception in GWVs with CMP without exacerbating pain symptoms or reducing lifestyle physical activity performed outside the exercise program (Stegner et al., 2021). However, neither the Donta 2003 nor Stegner 2021 studies explored the potential role of any plausible neurobiological mechanisms (e.g., exercise-induced neurotrophic factors; reduction in inflammatory mediators; improved pain regulation; changes to neuroanatomical structure) in the production of the treatment effects (Palmer et al., 2023; de Zoete et al., 2020; Herring and Meyer, 2024; Herold et al., 2019; Yarrow et al., 2010; Cuyul-Vásquez et al., 2023)—a critical need for translating and targeting potential therapies for larger-scale clinical use. The purpose of this study was to examine brain structural adaptations to 16 weeks of RET in GWVs with CMP and to test associations among brain structure, musculoskeletal strength, and symptoms. Brain structural outcomes were assessed using (i) diffusion tensor imaging (DTI) to measure WM tissue microstructure and (ii) voxel-based morphometry (VBM) of T1-weighted images to measure GM tissue volume. We hypothesized that RET would improve pain, alter WM microstructure in directions suggestive of better WM health, and increase GM volume compared to a waitlist-control (WLC) condition. We also hypothesized that changes in WM microstructure and GM volume would be associated with changes in strength and symptoms.

## Materials and methods

### Research design and methods overview

This study was part of a randomized controlled trial examining the influence of a 16-week RET program on disease perception and pain symptoms, physical activity, and brain structure and function in GWVs with CMP compared to a WLC group (ClinicalTrials.gov Identifier: NCT01350492).^1^ The overall goal of the trial was to determine whether RET is a safe and potentially efficacious treatment for GWVs with CMP. Details regarding the outcomes related to the primary aims and methods, and including the CONSORT flow diagram, have been previously described (Stegner et al., 2021). The specific focus of this investigation was to determine the influence of RET on cerebral WM microstructure and GM volume in GWVs with CMP. Secondary aims included examining the relationships between pain symptoms, strength adaptations, and brain structural outcomes. The procedures and methods described below detail the RET program, structural neuroimaging, and symptom assessments relevant to the current studies aims.

### Participants, screening, and inclusion criteria

Fifty-four U.S. military Veterans of the Persian Gulf War (1990–1992) with chronic widespread musculoskeletal pain (ages 40–64 years) were recruited and randomly assigned to 16 weeks of RET or WLC. Enrollment for this project was initiated in July 2013 and continued until May 2018. The participant ages reported in the present manuscript correspond to their age at the time of enrollment. Chronic widespread pain was operationally defined as current pain in multiple (≥3) body regions that was present for at least 3 months. Body regions were designated as head, axial spine, right upper body, left upper body, right lower body, left lower body, upper back, and lower back. The widespread nature of the pain also had to have occurred post-deployment to the Persian Gulf War. All participants were recruited via established VA mechanisms (Stegner et al., 2021), initially screened by phone, and asked to provide a letter from their physician approving their participation in the case of assignment to the RET program.

### Exclusion criteria

Those who regularly engaged in resistance exercise were ineligible. Participants were screened to ensure that they were not disabled to the point where whole-body RET would not be possible (e.g., wheelchair bound). Potential participants were further screened and excluded for the following: presence of a medically defined pain condition (e.g., rheumatoid arthritis) which could explain the majority of their chronic pain, use of anti-convulsant or narcotic analgesic medication within the 3 weeks prior to enrollment, major depressive disorder with melancholic features, substance abuse or dependence (within 2 years), schizophrenia, and bipolar disorder. As the present investigation included MRI scans with a cognitive task and thermal pain testing, individuals with contraindications for MRI (e.g., claustrophobia, embedded shrapnel, implanted medical device), colorblindness, or peripheral neuropathies/skin rashes affecting the pain test area (i.e., left palm) were also excluded. Participants were also assessed for the presence of FM, using the 2010 American College of Rheumatology criteria (Wolfe et al., 2010), although FM status was not exclusionary.

### Procedures overview

Following written informed consent, participants underwent three phases of testing that included: (1) a mental health interview (Lecrubier et al., 1997) and a clinical assessment at the Madison VA hospital to verify eligibility; (2) 16 weeks of RET or WLC depending on group assignment; and (3) MRI brain imaging at six time points. After completing the initial test day, participants were randomly assigned to their condition, either RET or WLC, and scheduled for the baseline MRI scan. Participants were randomly assigned in blocks to ensure an equal number in each group using the Research Randomizer tool (randomizer.org).

Prior to each MRI test session, participants were reminded not to consume coffee or other food or drinks containing caffeine for at least 4 hours, not to smoke or use tobacco or nicotine products for at least 4 hours, and not to consume alcohol, take any additional medications such as aspirin or other non-steroidal anti-inflammatory medications, cold medicine or antihistamines for at least 24 hours prior to testing. Participants were reimbursed for travel expenses at the standard VA rate up to a maximum reimbursement of $750 and received $75 for each testing session.

### Behavioral measurements

As part of their MRI scan days, participants filled out multiple questionnaires. Those that are pertinent to this investigation included: (1) Demographic information, (2) Short-form McGill Pain Questionnaire (SF-MPQ; Melzack, 1987), (3) Fibromyalgia Impact Questionnaire (FIQ; Burckhardt et al., 1991), (4) Multi-dimensional Fatigue Inventory (MFI; Smets et al., 1995), (5) The Veterans Health Survey SF-36 & Health Behaviors (VR-36; Hays et al., 1993), (6) Profile of Mood States (POMS; McNair et al., 1971), and (7) International Physical Activity Questionnaire (IPAQ: Craig et al., 2003). The symptom surveys have well-established evidence to support their validity and reliability and have been standards in the field of chronic pain research.

Both groups also completed weekly questionnaires during the 16-week intervention period. Those in the WLC group were asked to complete an at-home questionnaire each week that included the SF-MPQ, POMS, and a visual analog scale (VAS) of their current fatigue. Those in the RET group completed questionnaires in-person before and after each RET session. Each of these questionnaires included the SF-MPQ and the fatigue VAS. The POMS was included in at least one of the pre-exercise questionnaires each week. The SF-MPQ and the fatigue VAS scale always asked the participants about how they were feeling “right now.”

### Magnetic resonance imaging

Participants completed a baseline MRI scan that included all brain structural measurements including a high resolution T1-weighted image and DTI. During the trial, participants from both the RET and WLC conditions were scheduled to return to the Waisman Center brain imaging facility on weeks 6, 11 and 17 of the intervention for repeat brain imaging scans. Scanning timepoints during training were chosen to be evenly spaced apart and to assess whether any changes in brain outcomes corresponded with either the exercise training progression or symptom changes. Follow up scans at 6- and 12-months post-intervention were also completed to determine whether any treatment effects lasted beyond the intervention period. For the RET group, the testing was conducted on the non-exercise training days or prior to exercise on training days to avoid having acute exercise influence the brain imaging outcomes.

### Neuroimaging data acquisition parameters

High-resolution anatomical MR images were acquired at 3 Tesla (GE Discovery MR750, GE Medical Systems, WI) using an 8-channel whole-head coil, foam cushions to restrict head motion, and MRI compatible headphones for communications from the experimenter and to minimize scanner noise. The primary brain outcomes for the present study are GM volume assessed using VBM of the T1-weighted scans and WM tissue microstructure assessed by DTI.

Anatomical volumes were acquired using T1-weighted images (3D IR-prepped fast-gradient echo-pulse sequence) with the following parameters: 160 (1 mm thick) axial images, 256 × 256 × 160 matrix, Repetition Time = 8.16 ms, Echo Time = 3.18 ms, Inversion Time = 450 ms, Field of View = 25.6 cm.

Diffusion-weighted images were acquired with a single shot spin-echo echo-planar imaging pulse sequence, using 60 optimum non-collinear encoding directions (obtained by minimum energy numerical optimization) with a diffusion weighting of 1,000 s/mm^2^ and eight non-diffusion weighted reference images. Other imaging parameters were: Echo Time = 78.2 ms, Repetition Time = 8,000 ms 1, NEX (magnitude averaging) = 1, and an image acquisition matrix of 128 × 128 over a field of view of 240 × 240 mm^2^. The cerebrum was covered in 74 contiguous 2-mm thick axial slices. The total acquisition time was roughly 9 minutes. High-order shimming was always performed prior to the DTI acquisition to optimize the homogeneity of the magnetic field across the brain and to minimize echo planar image distortions. A main magnetic field inhomogeneity map was additionally estimated via the IDEAL fat/water estimation technique (Reeder et al., 2005).

### Intervention period: 16-week resistance exercise training program

The RET program was standardized, progressive, and individualized to the participants’ initial capacity; but sensitive to the potential for symptom exacerbation in GWVs with CMP. The program was designed to elicit a training effect (i.e., strength gains) for all adherent and able participants, but was conducted at a relatively low intensity to reduce the potential for symptom exacerbation. The overall design, rationale, feasibility, efficacy, and safety of the study have been previously described (Stegner et al., 2021).

Participants assigned to the exercise condition were asked to complete 90-minute resistance training sessions twice per week for 16 weeks in a resistance training facility created specifically for this study at the Madison VA hospital. RET was conducted on an individual basis with an exercise specialist to minimize potential therapeutic benefits that might occur as a result of social interaction with group exercise (Puetz et al., 2006). Published methods were employed to instruct participants on how to perform resistance exercise (Baechle and Groves, 1994). Very light loads were used during the first week to allow participants to learn proper breathing and lifting techniques, and habituate to the exercise. Complete details of the exercise training progression are published in Stegner et al. (Stegner et al., 2021).

### Exercise prescription

All training sessions began with a 5-minute warm-up session on a recumbent stationary bike. The intensity was intentionally low and selected by the participant. In accordance with published guidelines (Pollock et al., 2000; ACSM position stand),^2^ participants completed 10 resistance exercises targeting the whole body, which included: leg press, chest press, leg curl, shoulder press, leg extension, lat pull-down, back extension, abdominal crunch, arm curl, and arm extension. An estimated one-repetition maximum (1RM) procedure (based on a five-repetition max and the National Academy of Sports Medicine conversion chart)^3^ was used (RET group only) to determine the initial training loads (with the exceptions of abdominal and back extension lifts) and to track strength progression for RET (Stegner et al., 2021; Dohoney et al., 2002; Mayhew et al., 2008; Reynolds et al., 2006). Initial resistance for the intervention training sessions was set to 25% of the 1RM for upper body exercises, and 35% of 1RM for lower body exercises. Following a warm-up at a very low intensity, participants completed two sets of 10–15 repetitions for each exercise with 1 minute of rest between sets and 2 minutes of rest between exercises.

After the first 2 weeks, the load was increased as tolerated by participants. The goal was to complete 7–10 exercises during each RET session. All 10 exercises were done at least once per week (i.e., if participant did not complete a particular exercise during the first session that week, they performed it during the second session), thereby allowing for safe, progressive muscular adaptations to occur over the 16-week experiment (Jones, 2015; Larsson et al., 2015).

### Intervention period: wait-list control procedures

The WLC group followed the same MRI test day schedule as the RET group, however, they received no intervention and filled out weekly questionnaires from home (SF-MPQ, Fatigue VAS, POMS). They also completed the MRI scanning at the same time intervals as the RET group.

### MRI data processing

#### T1-weighted imaging

Pre-processing of the T1-weighted images included a collection of steps to clean, segment, register, and modulate data to prepare for VBM analyses. Files were converted from DICOM (Digital Imaging and Communications in Medicine) format to NifTi (Neuroimaging Informatics Technology Initiative) format using the dcm2nii tool (Li et al., 2016). Following conversion, images were bias field corrected via the N4 algorithm with an automated command from the Advanced Normalization Tools (ANTs) package (Tustison et al., 2010). Non-brain tissue was then removed for all images using the FMRIB Software Library’s (Jenkinson et al., 2012) brain extraction tool (Smith, 2002). Images were then reviewed to ensure complete removal of all non-brain tissue, no errant removal of any brain tissue, and a general data quality check (e.g., motion artifact). The following processing procedures were carried out in Statistical Parametric Mapping (SPM) 12 using the Diffeomorphic Anatomical Registration Through Exponentiated Lie Algebra (DARTEL) processing pipeline (Ashburner, 2007). First, native space images underwent GM, WM, and cerebrospinal fluid segmentation using the SPM Computational Anatomy Toolbox (CAT 12) Segment Longitudinal Data tool (Gaser et al., 2022). The GM and WM images were then brought into rough alignment between all subjects (DARTEL space) and averaged. The GM and WM images of each subject were then registered to an average image of the entire sample with the flow fields preserved. The flow fields were then used to improve the registration of the GM images to Montreal Neurological Institute (MNI) 152 space. Following registration to MNI space, all images were modulated with their Jacobian determinants. Images were smoothed with 8-mm isotropic gaussian kernel. An explicit whole brain GM mask was created from the MNI template by binarizing the MNI image. The SPM “get totals” tool was used to calculate total and regional GM volumes. Total brain volume was calculated by totaling the GM, WM, and cerebrospinal fluid volumes.

### Diffusion tensor imaging

Data were processed using an in-house processing pipeline from the Diffusion Imaging in Python (DIPY) toolkit (Garyfallidis et al., 2014), FMRIB Software Library (FSL) v6.0.3 (Jenkinson et al., 2012), Analysis of Functional Neuroimages (AFNI; Cox, 1996), and MRtrix (Tournier et al., 2012) software packages.

Processing included removal of Rician noise (Veraart et al., 2016), removal of Gibbs ringing artifact (Kellner et al., 2016), correction for eddy-currents and motion using FSL’s EDDY tool (Andersson and Sotiropoulos, 2016) with the outlier detection and replacement (Andersson et al., 2016) option (‘repol’) to flag volumes for removal of severe artifacts (Andersson et al., 2016). The tool flags slices within each diffusion-weighted image volume where intensities are four times lower than Gaussian estimates. To control for effects of severe motion, diffusion weighted image volumes with more than 15% of slices flagged as outliers, were removed before construction of the diffusion tensor. Non-parenchyma signals were removed using the FSL’s brain extraction tool (BET; Smith, 2002).

To correct for susceptibility distortions (e.g., stretching), an approach similar to the DR-BUDDI (Diffeomorphic Registration for Blip-Up Blip-Down Diffusion Imaging) method (Irfanoglu et al., 2015) was employed. With this method, diffusion-weighted images were nonlinearly aligned to the structural T1-weighted image that had been down-sampled to the native diffusion weighted image resolution. This approach was used in place of a fieldmap-based correction as the fieldmaps that were acquired were corrupted.

Diffusion tensors were estimated for each voxel using a weighted least squares fitting algorithm as part of the DIPY (Garyfallidis et al., 2014) open software package. Eigenvalues (λ1, λ2, λ3) were calculated from these voxel-wise estimates of the diffusion tensor, and quantitative maps of fractional anisotropy (FA), mean diffusivity (MD), radial diffusivity (RD), and axial diffusivity (AD), were derived (Basser and Pierpaoli, 1996).

Visual quality checking of the images occurred at various points along the processing stream. Prior to spatial normalization, FA files were quality checked for major artifacts and abnormalities, such as striping, hyperintensities, lesions or missing WM, chopping, noise, anterior echo planar image distortions, and masking errors.

### Image registration and spatial normalization

Due to the need to resolve small microstructural changes, accurate image registration and volume normalization was a priority. Subject-specific FA templates were generated from data from all six timepoints using ANTs (Avants et al., 2009; Avants et al., 2011), which was then used to generate a population-specific template. The ANTs package was then used to register the FMRIB 1 mm FA template (available in FSL) to the population-specific template. Subject specific and population space warps were applied to the native space DTI measures, bringing the DTI measures into alignment with the template.

### TBSS skeleton creation

Tract-based spatial statistics (TBSS) were then applied to generate a skeleton from the mean FA map and subject specific regional maximum FA values were projected onto the group skeleton for statistical analyses as described by Smith et al. (Smith et al., 2004; Smith et al., 2006). Tract-based spatial statistics reduces the issues of poor intersubject registration and increases statistical power by reducing the number of statistical tests to the skeleton. MD, RD, and AD images were similarly projected onto the group skeleton.

### Statistical analyses

#### Demographics and baseline measures

Statistical analyses for demographic and baseline behavioral data were conducted using SPSS Statistics (v. 25, IBM, Armonk, NY). Demographic and baseline behavioral data were compared between the RET and WLC groups using independent Student’s t-tests and chi-squared tests where appropriate. Effect sizes were estimated using Hedges’ g, Cramer’s V, and 95% confidence intervals. The level of significance was set to an alpha of 0.05.

#### Behavioral data analyses—pain, fatigue, and mood responses

##### Between group analyses

Linear mixed effects models were used to test the effects of RET on pain (SF-MPQ total), fatigue (0–100 VAS), and mood [POMS Total Mood Disturbance (TMD)] using the lme4 package in R (Bates et al., 2015). For these analyses, questionnaire data that were collected at baseline and prior to the first day of each week of training (i.e., data for each week of training) were used for the RET group. For the WLC group baseline and weekly questionnaire data were used. Group, Time, and the Group*Time interaction were entered as fixed factors with subject and slope modeled as random factors with alpha at 0.05. Default settings were used which employed the maximum likelihood method of estimation, an unstructured covariance matrix, and the Satterthwaite approximation for degrees of freedom.

##### Within-group (RET): associations between strength and symptoms

A linear regression model was used to examine the relationships between change in strength or total training volume and change in pain symptoms for the RET group only. Age was entered into the model as a covariate. Pain symptoms were defined as change in the pain VAS from the FIQ [i.e., Pain-VAS (0–100)] from baseline to the post-intervention time point (week 17), which aligns with their final MRI scan. The FIQ Pain-VAS was chosen because it asked individuals to average their pain symptom intensity over the last week and was thus considered an appropriate estimate of their usual pain during the RET intervention. The independent variable for strength was the percent change in estimated 1RM values in pounds (relative to body weight in pounds) from baseline to the final strength measurement timepoint at week 16 or the total training volume expressed as tonnage. The estimated 1RM values were averaged across the eight exercises for which 1RM values were obtained. The tonnage values were calculated as ‘Tonnage Per Day Relative to Body Weight’ = {[*Σ*(weight*reps_warmup_ + weight*reps_set1_ + weight*reps_set2_)/(Body Weight in kg)]} and values represented the first day of training minus the last day.

#### Neuroimaging analyses

All neuroimaging analyses were conducted within R (R Core Team, 2021) with the use of ANTs for R (ANTsR; Avants et al., 2008), an adaptation of the ANTs program designed to leverage the powerful statistical computing and visualization of R for the analysis of MR data. All 3D MR images for each imaging modality were combined into a singular 4D file with the FSLmerge tool which were then read into R using ANTsR. Age was included in all neuroimaging statistical models as a covariate of non-interest and total brain volume was additionally included as a covariate of non-interest for analyses of GM only. False Discovery Rate (FDR) corrections at an alpha of 0.05 were applied to all analyses. Intraclass correlation coefficients were calculated for nine GM volume regions (Amygdala, Caudate, Hippocampus, Insula, Mid-Cingulate Cortex, Post-Central Gyrus, Pre-Central Gyrus, Putamen, and Thalamus) and the four DTI metrics (FA, MD, RD, RA) across the six scan test days (baseline, week 6, week 11, week 17, 6-months follow up, 12-months follow up).

##### Changes in WM and GM over the intervention period

Separate tests were conducted for each dependent variable of interest, specifically GM volume and each DTI metric (FA, MD, RD, AD). Linear mixed effects models for each outcome tested the effect of RET on GM volume and WM microstructure over time using the lme4 package (Bates et al., 2015). Group (RET vs. WLC) and scan time point (days since start of intervention) were entered as the fixed factors along with the group by time interaction; subject and slope were modeled as random factors. Default settings were used which employed the maximum likelihood method of estimation, an unstructured covariance matrix, and the Satterthwaite approximation for degrees of freedom. Analyses were conducted for the full trial including four scans during the 16-week training period and two scans at 6- and 12-month follow-ups; as well as only the four scans that occurred during the 16-week training period.

##### Within-group (RET) associations between WM, GM, strength, and pain symptoms

Separate linear mixed effects models were used to examine the relationship between change in pain symptoms or change in strength and change in brain structure over the course of the 16-week intervention. These exploratory analyses were only conducted for GWVs in the RET group. As with the neuroimaging analyses, linear mixed effects models were used and either strength (training volume and estimated 1RM) or pain (FIQ Pain-VAS 0–100) were added as covariates. For training volume, the week that corresponded to the date of the neuroimaging scan was used as the variable of interest. For pain symptoms, questionnaire data that were collected prior to the neuroimaging scan were used. As with the between-group analyses, data for the full trial and the 16-week training period were tested separately.

## Results

Our previous paper (Stegner et al., 2021) described the overall safety and efficacy of the trial intervention and follow up period, showing that GWVs with CMP assigned to RET reported improvements in their disease perception (i.e., Patient Global Impression of Change scores) without symptom exacerbation or reductions in total physical activity. To provide context for the neuroimaging analyses, select sample characteristics, RET details, and additional response variables pertinent to these analyses are described here as well.

### Demographics and baseline characteristics

From the initial sample of 54 GWVs, 8 individuals (RET: *n* = 4, WLC: *n* = 4) withdrew or were lost to follow-up prior to baseline data collection and the start of the study. As a result, their data were excluded from subsequent analyses. For the remaining 46 participants (RET: *n* = 24, WLC: *n* = 22), 37 completed all four MRI scans during the intervention resulting in an attendance rate of 80%. Subsequently, 31 participants completed all six MRI scans.

Groups were not statistically different on demographics, symptom, diagnostic, or mood measurements at baseline (*p* > 0.05; Table 1).

**Table 1:** Demographics and baseline symptom characteristics.

	RET (*n* = 24)	WLC (*n* = 22)		
	Mean	Mean	Effect Size	*p*-value
Age, years	50.0 (7.2)	49.5 (5.2)	*g* = 0.08	0.79
Height, cm	174.4 (7.6)	175.1 (9.6)	*g* = −0.08	0.78
Weight, kg	94.6 (15.9)	92.0 (12.7)	*g* = 0.18	0.54
BMI	31.1 (4.6)	30.1 (4.5)	*g* = 0.21	0.48
Sex, % female (n)	21% (5)	14% (3)	*V* = 0.10	0.52
Race, % minority (n)	8% (2)	27% (6)	*V* = 0.25	0.24
Education, % college or postgrad degree (n)	50% (12)	55% (12)	*V* = 0.28	0.60
MDD Diagnosis, % (n)	8% (2)	9% (2)	*V* = 0.00	1.00
PTSD Diagnosis, % (n)	4% (1)	9% (2)	*V* = 0.09	0.55
GAD Diagnosis, % (n)	8% (2)	0% (0)	*V* = 0.22	0.15
FM Diagnosis, % (n)	33% (8)	41% (9)	*V* = 0.08	0.60
WPI Score	6.6 (2.7)	7.2 (3.8)	*g* = −0.18	0.54
Symptom Severity Score	5.3 (2.9)	5.6 (2.6)	*g* = −0.09	0.75
SF-MPQ-Total	7.9 (6.0)	8.2 (7.2)	*g* = −0.04	0.89
FIQ-Pain VAS	4.7 (2.5)	3.5 (2.4)	*g* = 0.48	0.11
FIQ-Total	30.6 (14.7)	28.1 (12.8)	*g* = 0.32	0.28
VR-36-Bodily Pain Score	47.7 (17.2)	53.1 (16.0)	*g* = −0.32	0.29
VR-36-Physical Health Component Score	40.2 (8.6)	44.7 (7.9)	*g* = −0.54	0.08
VR-36-Mental Health Component Score	47.8 (7.4)	47.7 (6.7)	*g* = 0.01	0.97
MFI-Total	57.0 (15.2)	56.1 (15.1)	*g* = 0.06	0.85
POMS-Total Mood Disturbance	129.6 (27.1)	124.7 (27.4)	*g* = 0.18	0.55
IPAQ-Total	4634.4 (6056.2)	6080.1 (7466.3)	*g* = 0.21	0.15
IPAQ-Moderate	559.7 (696.1)	760.4 (930.2)	*g* = 0.24	0.14
IPAQ-Walking	466.3 (888.4)	487.8 (659.5)	*g* = 0.02	0.30
IPAQ-Vigorous	135.9 (198.8)	216.1 (363.1)	*g* = 0.27	0.21
IPAQ-Sitting	569.6 (518.2)	776.3 (831.3)	*g* = 0.30	0.45

Values are Mean (+/− SD) unless otherwise noted. Effect sizes are either Hedges’ g or Cramer’s V. For Hedges’ g, 0.2, 0.5, and 0.8 are described as small, medium, and large, respectively. For Cramer’s V, 0.1, 0.3, and 0.5 are described as small, medium, and large, respectively (Cohen, 1988). FM diagnosis, WPI score, and symptom severity score are based on the 2010 American College of Rheumatology criteria (Wolfe et al., 2010). BMI, body mass index; MDD, Major Depressive Disorder; PTSD, Post-Traumatic Stress Disorder; GAD, General Anxiety Disorder; FM, fibromyalgia; WPI, Widespread Pain Index; SF-MPQ, Short Form McGill Pain Questionnaire; VAS, visual analog scale; FIQ, Fibromyalgia Impact Questionnaire; VR-36, Veterans Health Survey SF-36; MFI, Multi-Dimensional Fatigue Inventory; POMS, Profile of Mood States; IPAQ, International Physical Activity Questionnaire.

### Adherence, compliance, and progression to the RET program

For the GWVs assigned to the RET condition (*n* = 24), the average number of training sessions attended, out of 32 possible, was 27.9 (+/− 5.8) or 87%. The RET group completed 79.3% of prescribed sets (1 set is defined as completing 10–15 reps) demonstrating good compliance with the protocol. An increase in training volume was observed for the RET group (Supplementary Figure 1). GWVs in the RET group increased their 1RM between 19% and 46% (*p* ≤ 0.001) for the eight lifts monitored, representing moderate to large strength gains for each exercise (Table 2).

**Table 2:** Estimated one-repetition maximums.

	Baseline	Week 16		
	Mean (SD)	Mean (SD)	*p*-value	Hedges’ *g*
Leg press	1.78 (0.38)	2.20 (0.52)	<0.001	0.98
Chest press	0.78 (0.19)	0.92 (0.26)	<0.001	0.64
Shoulder press	0.48 (0.16)	0.59 (0.19)	<0.001	063
Lat pulldown	0.73 (0.16)	0.88 (0.18)	<0.001	0.90
Arm curl	0.41 (0.11)	0.46 (0.09)	0.009	0.46
Arm extension	0.46 (0.15)	0.56 (0.14)	<0.001	0.72
Leg curl	0.64 (0.15)	0.74 (0.17)	<0.001	0.62
Leg extension	0.69 (0.26)	0.91 (0.26)	<0.001	0.85

Values represent the estimated one-repetition maximum (kg) for each lift divided by body weight (kg), averaged across the sample. Data from the RET participants only. Sample is limited to those GWVs that completed both the baseline and 16-week maximum testing (*n* = 21). Baseline to week 16 comparisons conducted with Paired Samples *t*-tests. Effect sizes are Hedges’ g and 0.2, 0.5, and 0.8 are described as small, medium, and large, respectively.

### Pain, fatigue, and mood responses and associations

**Figure 1:**
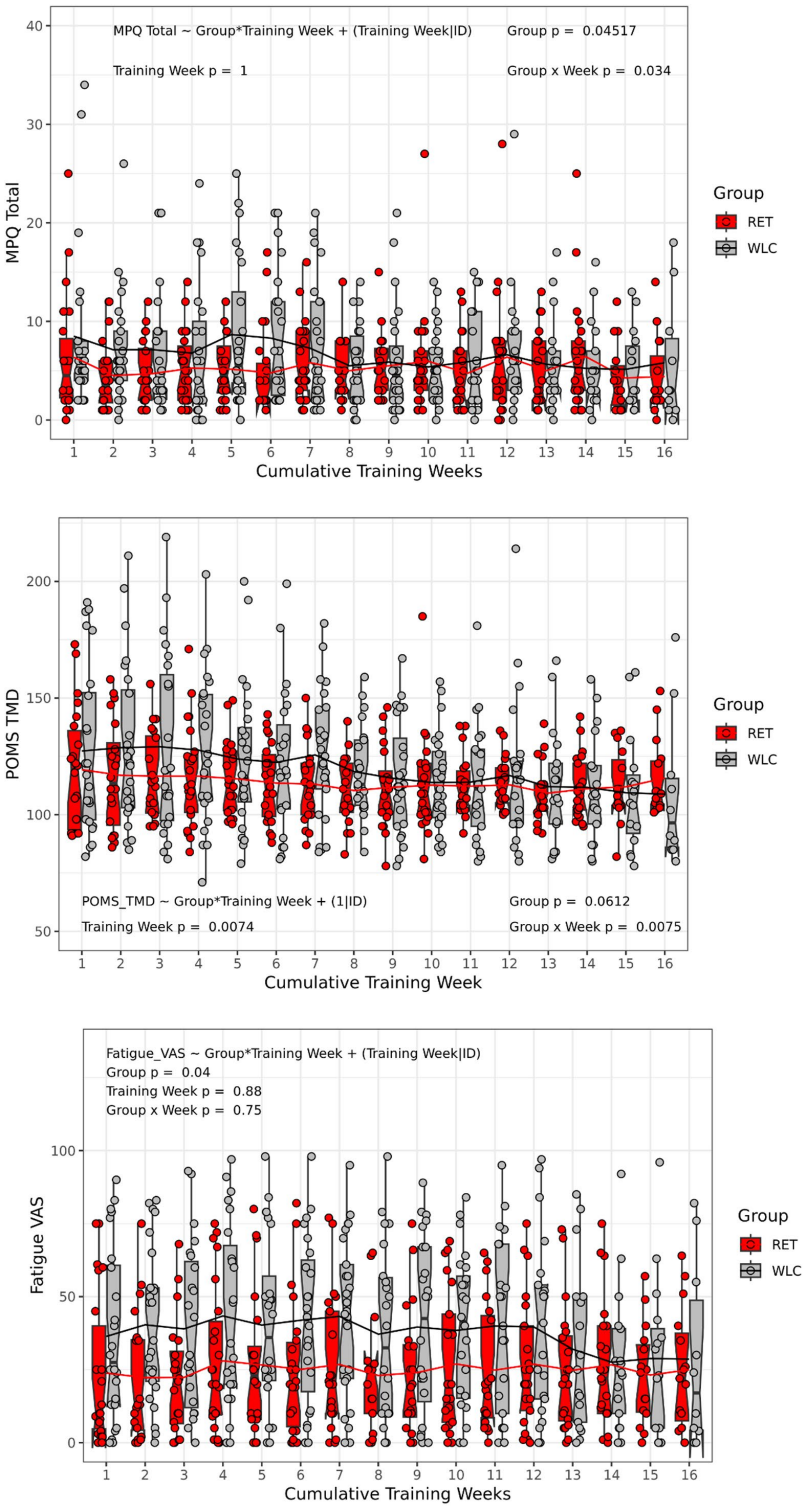
Pain, fatigue, and mood responses. Box and whisker plots with individual scatterplots and mean trend lines overlaid representing weekly pain (MPQ total), fatigue (0–100 VAS), and mood (POMS TMD) in response to 16 weeks of RET or WLC. The height of the box (Red = RET, Grey = WLC) is the interquartile range (IQR) and the whiskers are 1.5 * IQR. The solid black line in the middle of the box is the median value for each group and week of training and the notches represent the 95% confidence interval. Note that only the RET group were actually doing weekly resistance trainings coinciding with the administration of symptom questionnaires, but the WLC were still completing them at all time points at their home. MPQ, Short form McGill Pain Questionnaire; VAS, visual analog scale; POMS TMD, Profile of Mood States: Total Mood Disturbance; RET, resistance exercise training; WLC, Wait list control.

There was a significant main effect of Group (*p* < 0.05) and a significant Group*Time interaction (*p* < 0.04) for MPQ total scores across the 16-week intervention period. There was a main effect for Group (*p* < 0.05), but no Time or Group*Time effects (*p* > 0.05) for fatigue. The main effect of Group was characterized by lower fatigue ratings for the RET group compared to the WLC group. For POMS TMD, there was a main effect of Time (*p* < 0.01), and a significant Group*Time interaction (*p* < 0.01). The main effect of Time was characterized by a decrease in TMD for both RET and WLC groups. These results are illustrated in (Figure 1).

**Figure 2:**
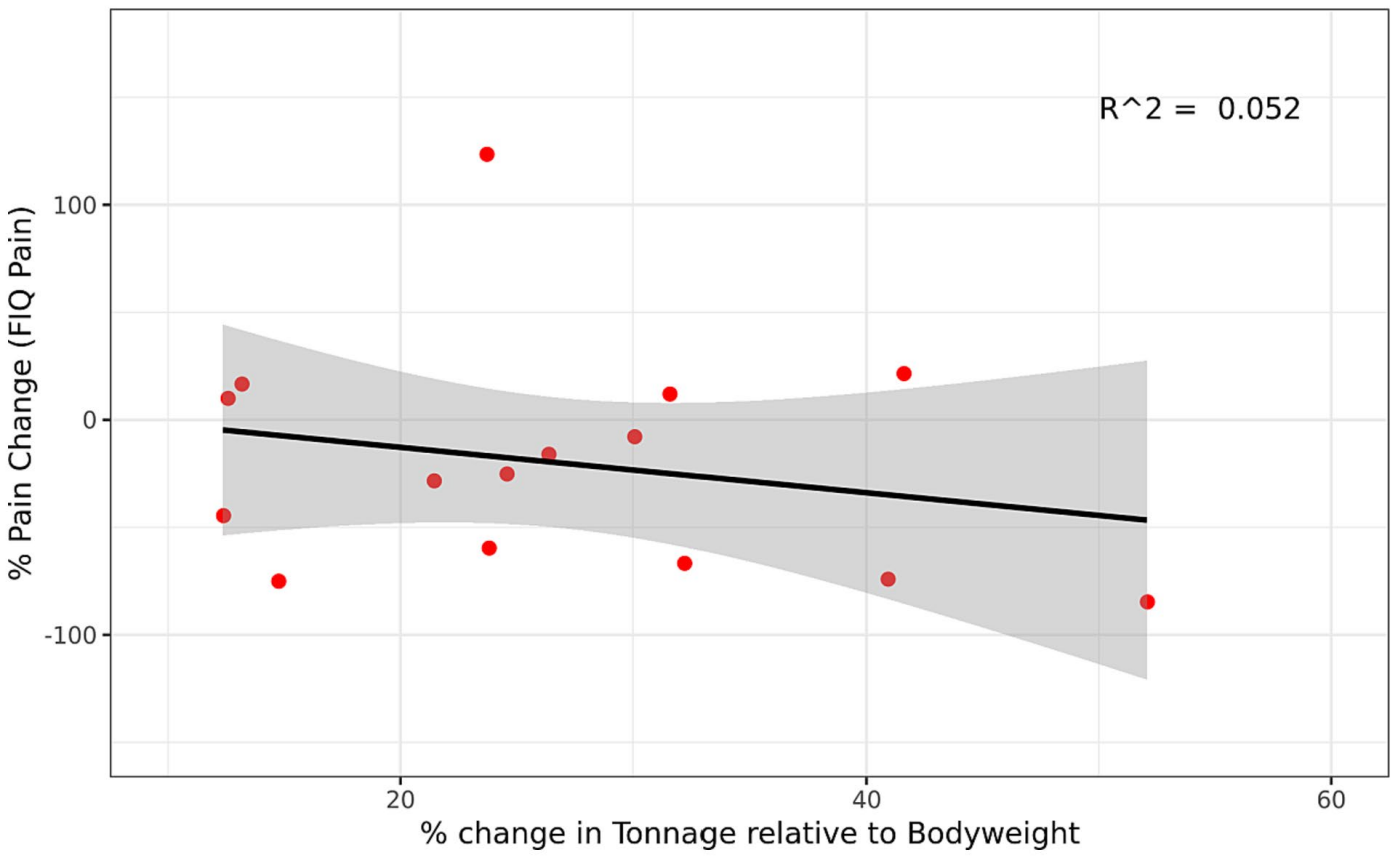
Relationship between Change in Strength and Change in Pain Symptoms in the RET group. Regression between percent change in strength (estimated 1RM) and percent change in pain symptoms (FIQ Pain-VAS) for the RET group. Percent change was for week 1 to week 16 and was calculated based on the equation [(Post-Pre)/Pre]*100. A positive percent change in strength represents an increase in strength and a negative percent change in pain represents a reduction in pain symptoms. Gray region represents 95% confidence interval. FIQ, Fibromyalgia Impact Questionnaire; VAS, visual analog scale; RET, resistance exercise training.

Changes in maximal strength from week 1 to week 16 were not significantly (*p* ≥ 0.05) associated with changes in pain symptoms (Figure 2). Likewise, changes in training volume were not significantly (*p* > 0.095) associated with changes in pain symptoms (Supplementary Figure 2).

### GM volume in response to RET

Intraclass correlations coefficients were greater than 0.85 (with the exception of the thalamus; with 0.73 for left thalamus and 0.77 for right thalamus) for the GM volume data across the six scanning visits.

There were no significant Group or Time main effects; nor was there a significant Group*Time interaction for either model (i.e., intervention period, 6- and 12-month follow up) following multiple comparison correction (*p* > 0.05). These data suggest that GM remained stable over time similarly between the RET and WLC groups. Uncorrected t-stat maps (Supplementary Figure 3) and effect size plots and table (Supplementary Figure 4; Supplementary Table 1) are presented in the Supplementary materials for data transparency purposes.

Neither strength nor pain were significantly (*p* > 0.05) associated with GM volume over the course of the trial.

### WM microstructure in response to RET

Intraclass correlations coefficients were greater than 0.90 for each of the DTI metrics (i.e., FA, MD, RD, AD) across the six scanning visits.

There were no significant main or interaction effects for WM microstructure for any DTI metric (FA, MD, RD, AD) or either model (intervention period, 6- and 12-month follow up) following multiple comparison correction (*p* ≥ 0.05). Very small clusters of non-contiguous voxels, representing less than 0.1% of all voxels, were identified by the main effect of Time. However, due to their minimal size and isolated nature, these findings were considered to be spurious. These data suggest that WM remained stable over time similarly between the RET and WLC groups. Uncorrected t-stat maps (Supplementary Figures 5–8) and effect size plots and table (Supplementary Figure 4; Supplementary Table 2) are presented in the Supplementary materials for data transparency purposes.

Neither strength nor pain were significantly (*p* > 0.05) associated with WM microstructure over the course of the trial.

## Discussion

The goal of this study was to determine the influence of 16 weeks of RET on GM volume and WM microstructure in GWVs with CMP, testing whether exercise training can stimulate neuroplastic changes. In addition, the relationship between changes in strength, symptoms, and brain structural measures were examined. Safety and efficacy were also essential elements of this training trial – both as proof of concept (i.e., the trial was successful in safely inducing moderate-to-large changes in strength) and to support the plausibility of observing brain changes due to the exercise stimulus (i.e., neural adaptations to strength training; Voss et al., 2013).

### Response to RET

Overall, this standardized RET program was safe for GWVs with CMP. This was demonstrated through two key pieces of evidence: (1) there were no serious adverse events (defined in Stegner et al., 2021) associated with the RET intervention, and (2) average pain, fatigue, and mood symptoms did not worsen. Efficacy was demonstrated through (1) moderate to large improvements in strength for the RET group and (2) a significant reduction in Veteran’s perceptions of their disease limitations (Stegner et al., 2021). Strength improvements were observed both through weekly strength progression and the estimated 1RM exercise performance. These data are consistent with clinical trials of RET for widespread pain in civilian populations such as FM that show average improvements in strength between 33% and 63% for interventions of 12–21 weeks in duration (Andrade et al., 2018; Busch et al., 2013). The Group*Time interaction for pain ratings is difficult to interpret as illustrated in Figure 1. Our interpretation is that the interaction is best characterized by a group separation from week 1 until week 7 and then the groups merge with no real differences in pain ratings. Thus, it is not clear whether pain improved for either group. The meaningful changes in strength observed in the exercise training group suggest that brain structural changes were biologically plausible (Voss et al., 2013).

Despite gains in strength, symptoms of pain and fatigue remained stable throughout the trial, with a decrease in mood disturbance over time for both groups. These data suggest that improved physical performance, of the magnitude observed in the current trial, was not sufficient to reduce symptoms associated with CMP in GWVs. Lack of symptom improvements may be due in part to the moderate levels of symptom burden at the start of the trial. Less than half of our sample [33 (RET)–41 (WLC)%] was diagnosed with FM and the average scores for both pain intensity [FIQ-Pain VAS = 4.7 (RET); 3.5 (WLC)] and impact [FIQ-Total = 30.6 (RET); 28.1 (WLC)] were relatively lower than other chronic pain populations. One large population-based survey of over 2,500 FM patients reported average pain intensities of 6.4 on a 0–10 scale (Bennett et al., 2007), while another showed average FIQ-Total scores averaging 63.0 (*n* = 721; Henriksen et al., 2009). However, based on the Veteran’s Health Survey our sample was similarly impaired in terms of bodily pain, physical health, and mental health compared to recent Veteran cohorts (Bodily Pain = 42–53, PCS = 42–55; Falvo et al., 2012) but slightly more impaired compared to the VA national averages (PCS = 35.2, MCS = 43.6; Kazis, 2000). Fatigue ratings (MFI = 56.1–57.0) were also lower than those reported by other fatiguing conditions such as ME/CFS (*n* = 292, MFI = 67.1), but similar to population estimates of chronically ill people who do not meet ME/CFS criteria (*n* = 269, MFI = 52.6; Lin et al., 2009). Lastly, few Veterans had a concurrent mental health diagnosis and had relatively low mood disturbance, with baseline values similar to non-clinical adult populations (Nyenhuis et al., 1999).

Additionally, the exercise dose may not have been intense enough to elicit improvements in pain. The average intensity achieved during the final 2 weeks of training ranged from 43–57% of 1RM, which is comparable to the initial prescription intensities (40–60%) used in trials of RET in FM (Rodríguez-Domínguez et al., 2024; da Silva et al., 2022). Considering those trials also included progression of their intensity, often ending around 70–80% of 1RM, our sample was consistently exercising at a lower intensity. Thus, while avoiding exacerbation of symptoms was a key goal of this study, the relatively low intensity of prescription may have resulted in more limited effects on pain.

### The effect of RET on brain structure

The mechanisms that underlie the effects of exercise in treating CMP have been inadequately investigated, are largely unknown, and likely involve multiple biological systems. We chose to focus on GM volume and WM microstructure because these measurements capture a range of neuropathological insults and because the effects of chronic pain on the brain are distributed throughout multiple regions (Filley, 2001; Jensen et al., 2016). GM contains neuronal cell bodies which are responsible for processing and transmitting information, while WM provides the interconnections between brain regions to create functional networks. Importantly, differences in GM volumes and WM microstructure have been shown to be associated with pain symptoms in GWVs with CMP (Ninneman et al., 2022; Van Riper et al., 2017). Moreover, GM volume and WM microstructure differences have been shown to occur across multiple brain regions in chronic pain (Gronemann et al., 2020; Smallwood et al., 2013). Combined, these results suggest that evidence of neural circuitry disruption could serve as a potential biomarker for chronic pain. Further, pain modulation and sensitivity are dysregulated in chronic pain (including among GWVs), as evidenced by enhanced facilitation, and reduced inhibition of responses to nociceptive stimuli (Cook et al., 2007; O’Brien et al., 2018; Staud et al., 2008). Therefore, if exercise were to alter GM volume and WM microstructure, it is plausible that pain modulation and sensitivity would improve and lead to reduced chronic pain symptoms.

Sixteen weeks of structured RET did not significantly change brain structure in GWVs with CMP. Across the whole brain, a differential response in GM volume or WM microstructure was not observed. Further, no significant relationships between changes in strength and either pain or brain structure were observed for the RET group. Brain structure was hypothesized to improve in response to changes in muscular strength and improvements in pain symptoms, thus, these results were counter to our hypotheses. The null findings are bolstered by the high degree of reliability for both VBM and DTI measures. We observed intraclass correlations of greater than 0.85 (except the thalamus) for VBM estimates of GM volume and 0.90 for each DTI metric. Thus, our null findings are not likely to be due to unreliable measurement of brain structure.

### Exercise-induced changes in gray matter volume

Numerous trials have tested aerobic and RET effects on GM volumes. Motivated by animal models showing neurogenesis of the hippocampal dentate gyrus of mice exposed to wheel running (van Praag et al., 1999), select human trials have reported increased brain volume following aerobic exercise training (Colcombe et al., 2006; Erickson et al., 2011); although it is important to note that the majority of aerobic exercise training trials have not reported brain volume increases, despite moderate improvements in cardiorespiratory fitness (Balbim et al., 2024). Further, these studies have been primarily focused on older adults and the prevention of cognitive decline. At least four studies have tested the effects of exercise training on brain volumes in chronic pain populations, including, knee osteoarthritis, chronic whiplash associated disorders, chronic spinal pain, and fibromyalgia (Liu et al., 2019; Murillo et al., 2024; Malfliet et al., 2018; Leon-Llamas et al., 2020). In knee osteoarthritis, 12 weeks of either stationary cycling or Tai Chi exercises reduced pain symptoms and increased the volume of the supplemental motor area (Liu et al., 2019). In chronic whiplash associated disorders, 3 sessions of pain neuroscience education, followed by 15 sessions of strengthening, aerobic, and coordination exercises reduced pain-related fear, anxiety, and hypervigilance and significantly increased the volume of the dorsolateral prefrontal cortex compared to a non-pain control group (Murillo et al., 2024). In chronic spinal pain, 12 weeks of pain neuroscience education combined with cognition-targeted motor control training reduced pressure pain sensitivity, central sensitization symptoms, and improved self-reported physical function, but did not alter brain morphometry (Malfliet et al., 2018). In fibromyalgia, there was no effect of an exergame program consisting of 24 weeks of balance, postural, and aerobic exercises on GM; however, fitness was positively associated with bilateral hippocampus and amygdala volumes (Leon-Llamas et al., 2020). These trials included several different exercise modalities (often combined) and focused on a region of interest approach in their GM volumetric analyses; and therefore, it is unclear if the results would be similar if the unbiased (i.e., voxelwise) approach used here was applied. Moreover, the studies by Liu et al., and Murillo et al., further constrained their analyses by only testing the volume of regions identified to be different from controls at baseline. Our analyses used an unbiased analysis of brain volumes and found no differences in response to RET. Our findings are in agreement with a recent meta-analysis of GM volumes in other neurodegenerative disorders (i.e., schizophrenia, multiple sclerosis, Alzheimer’s Disease) and two systematic reviews of exercise and CMP that reported no significant effect in any brain region following RET (Hvid et al., 2021) and insufficient evidence for exercise interventions in CMP (Cuyul-Vásquez et al., 2023), respectively.

### Exercise-induced changes in white matter microstructure

To date, few randomized controlled trials have measured the effect of exercise training on WM microstructure, and to the best of our knowledge none have involved individuals with chronic pain. Most trials in this small body of literature have used aerobic exercise training in older adults and have found mixed results. Some trials have reported improved WM microstructure in regions such as the fornix, uncinate, and superior longitudinal fasciculus (Burzynska et al., 2017; Krafft et al., 2014; Schaeffer et al., 2014; Mendez Colmenares et al., 2021) while others have reported no change (Polak et al., 2014; Voss et al., 2013; Fissler et al., 2017; Tarumi et al., 2020; Venkatraman et al., 2020; Clark et al., 2019). One previous trial examined RET and found a 2% decrease in MD in the corticospinal tract of healthy young adults. This trial involved 16 sessions, over 4 weeks, of maximum isometric contraction of the planter flexor (unilateral; Palmer et al., 2013), and was therefore quite different than our whole-body RET prescription. Positive effects on WM microstructure have been observed with motor skills training (Lakhani et al., 2016), but such tasks do not improve overall strength or physical function such as with traditional RET. Several important limitations could account for the lack of consistent findings among these studies. Most notably, different DTI methodologies were employed (e.g., tractography vs. voxelwise), different ROIs were explored, and a wide range of sample sizes (*n* = 4–49) were tested. In addition, considerable design and analysis limitations were present including failure to account for age and inconsistent reporting of exercise training compliance and attendance.

In the context of these previous studies, the lack of a significant effect of RET on brain structure does not provide much insight into whether exercise can treat pain through GM and/or WM mechanisms. Had we observed obvious changes in symptoms, we may have concluded that changes in brain structural health were not necessary for a treatment effect. Alternatively, had we observed improvements in brain structure but no changes in symptoms, we might have concluded that exercise improves brain health in CMP, but that these improvements are not specifically associated with pain processing. Although 16 weeks of progressive RET did not alter brain structure, given the small sample size, limited duration, and testing of a single low-to-moderate dose (Jones, 2015; Larsson et al., 2015), it is premature to fully discount RET’s potential role in treating chronic pain through a brain mechanism.

### Exercise training, central sensitization, and associated factors

Alternative explanations for the lack of obvious pain improvement could be that exercise training reduced central sensitization (i.e., heightened sensitivity and reduced modulatory capacity of the central nervous system) and/or systemic inflammation at a level that was not detectible by our chosen neuroimaging methods. There is mixed evidence for whether exercise training can reduce signs of central sensitization, measured via pressure-pain thresholds, in fibromyalgia (Belavy et al., 2021). Moreover, 12 weeks of aerobic exercise training did not affect brain responses to pressure stimuli (Micalos et al., 2014; Löfgren et al., 2023) or distraction-induced hypoalgesia (Martinsen et al., 2018). Acute exercise causes an increase in IL-6, but this is considered to act as a myokine and induce an anti-inflammatory cascade as opposed to the more typical proinflammatory signaling (Pedersen and Fischer, 2007). Regular exercise training is similarly associated with anti-inflammatory and neuroprotective effects via immunomodulation, activation of the antioxidant system, and reduction of adipose tissue (Scheffer and Latini, 2020). Although data concerning how exercise affects inflammation and chronic pain in humans are limited, aerobic exercise training in animal models of chronic constriction injury nerve pain show reductions of inflammatory markers including tumor necrosis factor alpha (TNF-*α*), interleukin one beta (IL-1β), and interleukin 6 (IL-6)—while increasing interleukin 10 (IL-10; Chen et al., 2012; Tsai et al., 2017). However, the type of exercise appears to matter; a recent meta-analysis of 59 studies examining the effects of resistance exercise training on chronic inflammation found reduced C-reactive protein (SMD = −0.28; 95% CI = −0.46, −0.10) but no significant effects on either TNF-α (SMD = −0.07; 95% CI = −0.31, 0.16) or IL-6 (SMD = −0.12; 95% CI = −0.31, 0.07; Rose et al., 2021). How central sensitization and/or inflammation and either brain structure or function interact with exercise training and treatment of CMP is to our knowledge, unknown.

### Limitations

The primary limitation of this study was the relatively small sample size. However, based on the longitudinal design of the study including brain structural outcomes at multiple (i.e., six) time points during training and longer-term follow-up (with high reliability), we are confident that our null findings were not due to lack of power to detect brain changes. An exploration of the effect sizes associated with the Group*Time interaction effects in our models revealed that only a very small minority of voxels exhibited even a moderate effect (Cohen’s *d* ≥ 0.5). These voxels were widely distributed in a seemingly unrelated manner for the GM results and even more rare and distributed in the WM outcomes. Given the above, we believe we were adequately powered to statistically identify moderate to large effect sizes due to differential group changes in GM and WM structure across time, which we generally did not observe.

This study focused specifically on GWVs with CMP and to reduce heterogeneity within our sample we employed a number of exclusionary criteria as well as exclusions related to the MRI environment. Although these strict procedures are a strength of our experimental design, they reduce generalizability to other chronic pain conditions and the wider spectrum of Gulf War illness. Finally, our dose of exercise may have been too low to affect brain structure. The trial was designed to ensure safety of our GWV participants and test whether exercise could be conducted without exacerbating symptoms—a significant concern of those with CMP. Future research using more intense and longer duration exercise prescriptions (i.e., larger exercise doses) will be necessary to test whether RET can affect brain structure in CMP (Herold et al., 2019).

It is important to also highlight that one limitation of DTI is that the diffusion model assumes that the diffusion of water follows a Gaussian distribution with a single directional maximum and is therefore only able to estimate a single fiber orientation within each voxel. This creates issues of partial volume averaging and therefore limited specificity regarding which microstructural characteristics may be responsible for potential diffusion changes. Therefore, if a unique combination of changes in axon diameter, count, and/or density as well as dendritic arborization modifications were to occur, DTI may be limited in its ability to detect an effect (Chen et al., 2018). In addition, non-neuronal cells, such as oligodendrocytes, may also play a role in diffusion characteristics (Chen et al., 2018). Newer diffusion imaging techniques such as neurite orientation dispersion and density imaging (NODDI; Zhang et al., 2012) improve upon the traditional DTI model and are more sensitive to such microstructural changes.

## Conclusion

Results from this randomized controlled trial support that 16 weeks of low-to-moderate intensity RET (i) improved musculoskeletal strength and (ii) did not exacerbate symptoms (i.e., pain, fatigue, and mood), but was (iii) insufficient to alter brain structure in GWVs with CMP.

## Data Availability

The raw data supporting the conclusions of this article will be made available by the authors, without undue reservation.

## Footnotes

^1^ https://www.clinicaltrials.gov/study/NCT01350492

^2^ http://www.exrx.net/WeightTraining/Guidelines.html

^3^ https://www.nasm.org/docs/pdf/cpt-1rm-conversion-chart.pdf?sfvrsn=562ff933_8
