# Supplementary material for: 16 weeks of moderate intensity resistance exercise improves strength but is insufficient to alter brain structure in Gulf War Veterans with chronic musculoskeletal pain: a randomized controlled trial
Source: Front Neurosci. 2025 Apr 16;19:1488397. doi: 10.3389/fnins.2025.1488397 (PMC12040992; doi:10.3389/fnins.2025.1488397)
Supplement: Supplementary file 3 [file Data_Sheet_1.docx]

Supplemental Materials

**Supplemental** **Figure 1.** **Training Volume Progression.** Average training volume at each session as a product of exercise sets, repetitions, and loads - relative to body weight (error bars represent standard error of the mean). The 26 sessions displayed only include prescribed intervention sessions and do to not include the 6 max testing assessment sessions. Note that exercises were limited to 10, sets were limited to 3 (including warm-up), repetitions were specified at 10-15 per set, pace was standardized between individuals, and range of motion was consistent with individuals for each exercise. The calculation for ‘Tonnage Per Day Relative to Body Weight’ = {[Σ(weight*reps_warmup_ + weight*reps_set1_ + weight*reps_set2_)_(ALL 10 EXERCISES)_]/(Body Weight in kg)].

**
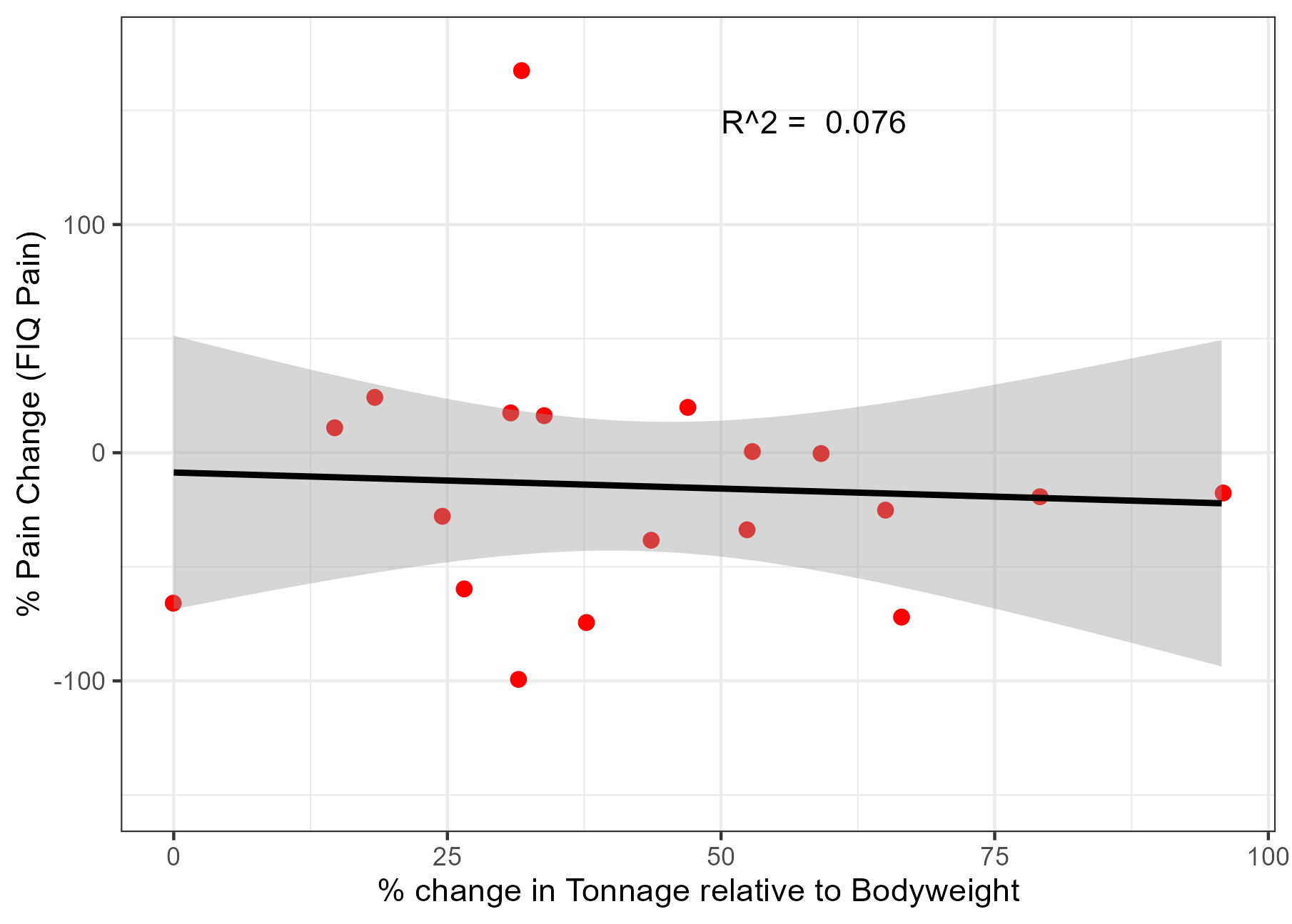
**

**Supplemental Figure 2.**

Association between percent change in pain (FIQ Pain VAS) and percent change in total training volume expressed as tonnage (Percent Tonnage Change /  BodyWeight). Values are for the first day of training minus the last day. The calculation for ‘Tonnage Per Day Relative to Body Weight’ = {[Σ(weight*reps_warmup_ + weight*reps_set1_ + weight*reps_set2_)_(ALL 10 EXERCISES)_]/(Body Weight in kg)].

Abbreviations: FIQ – Fibromyalgia Impact Questionnaire, VAS – Visual Analogue Scale


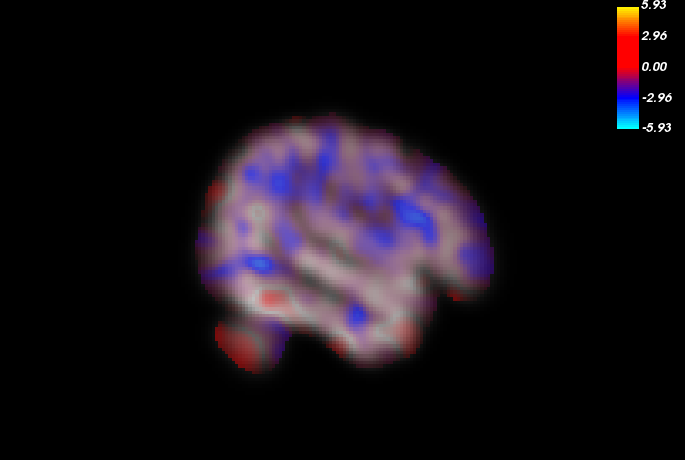

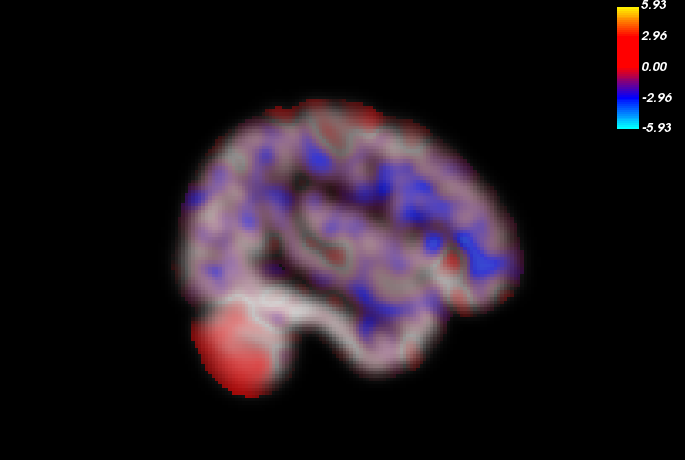

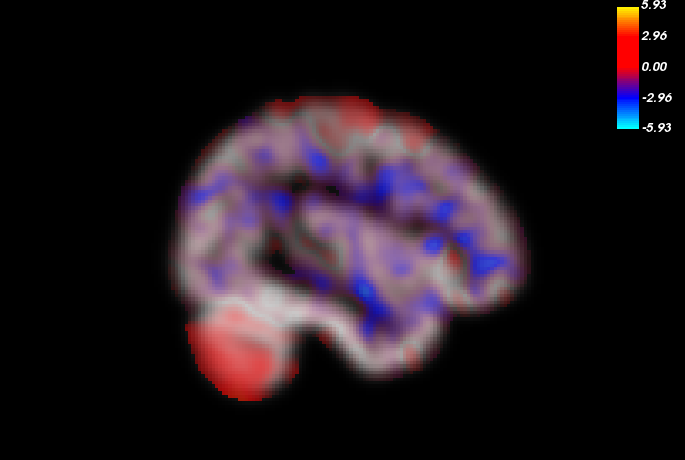

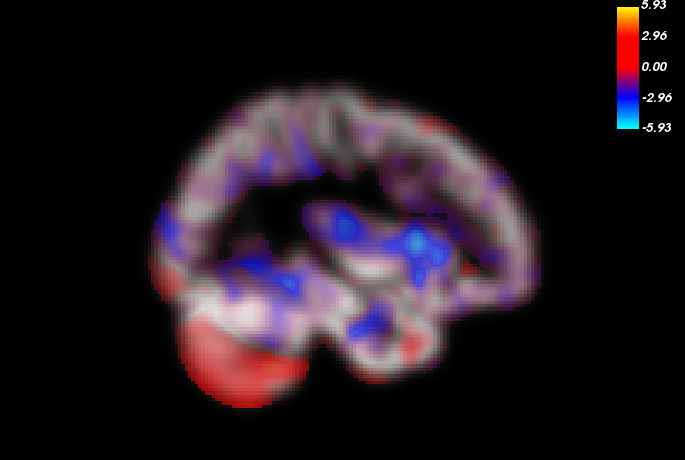

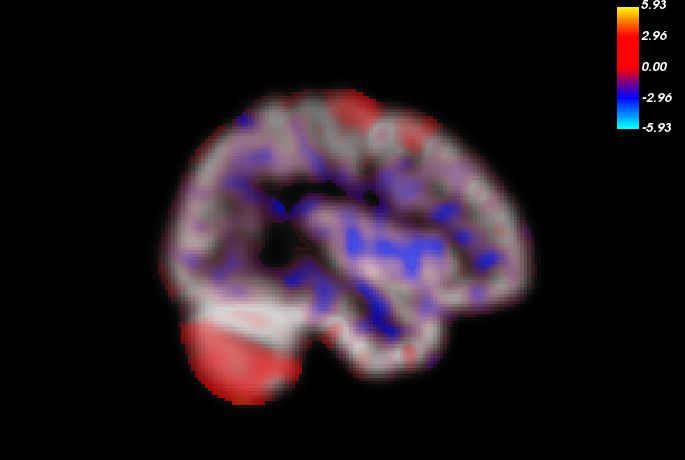

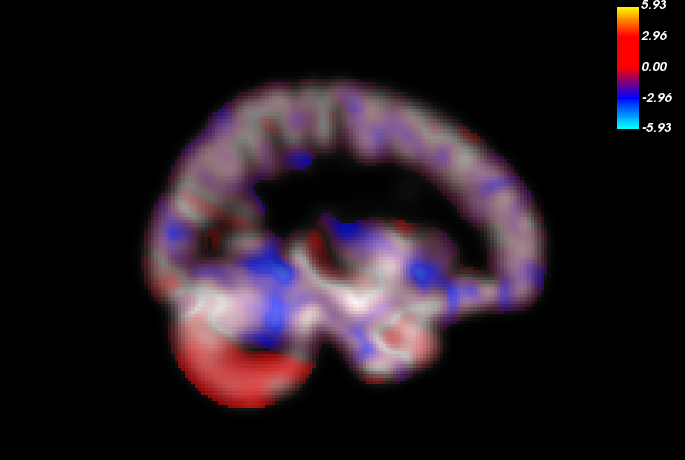

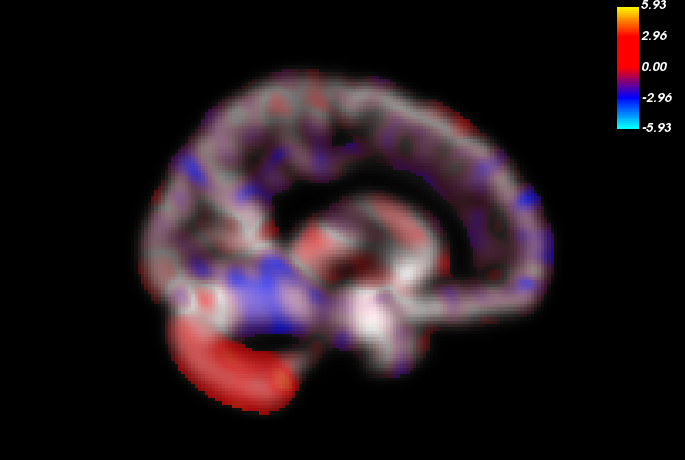

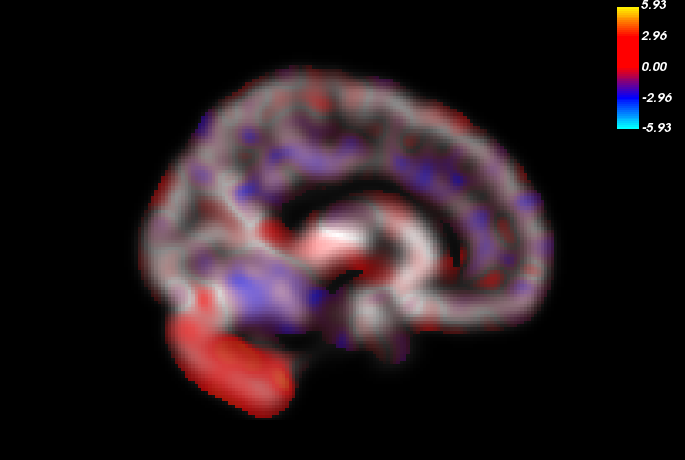

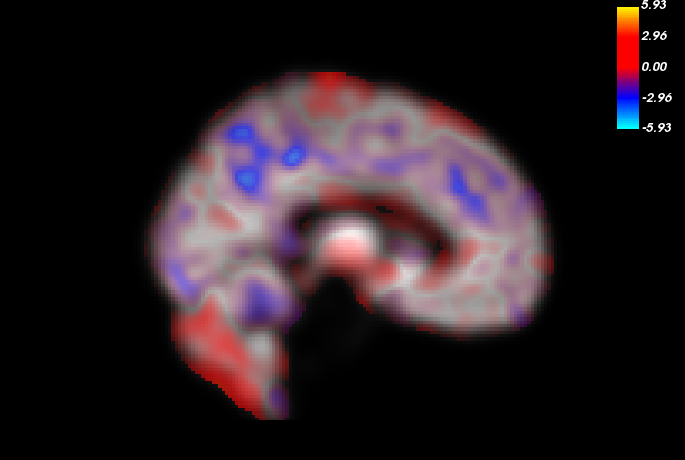

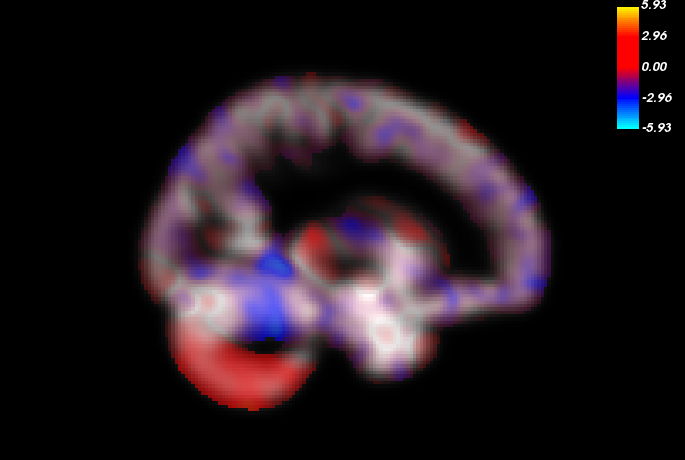

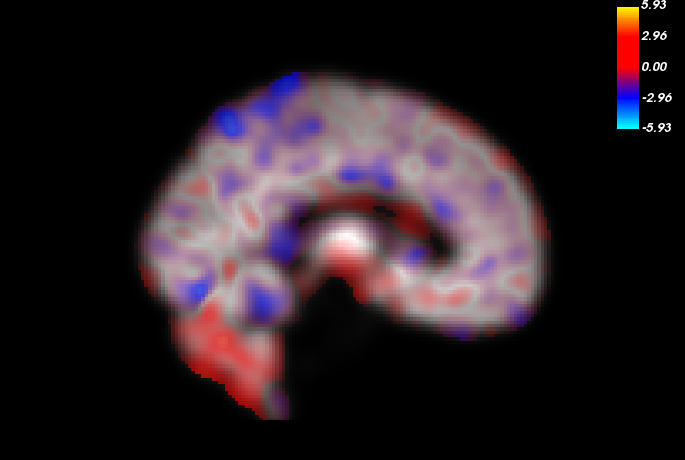

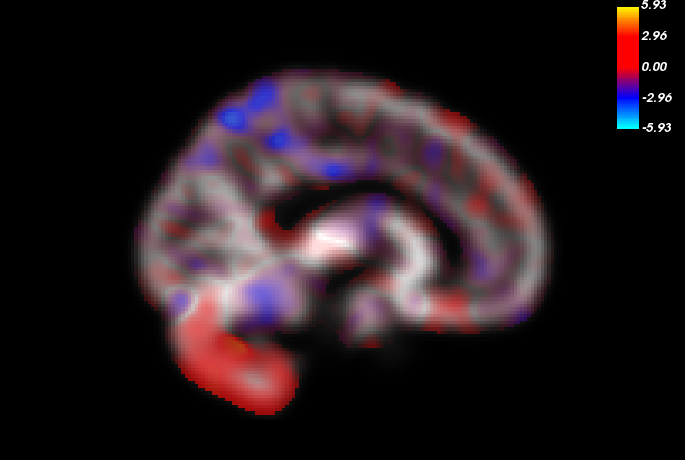

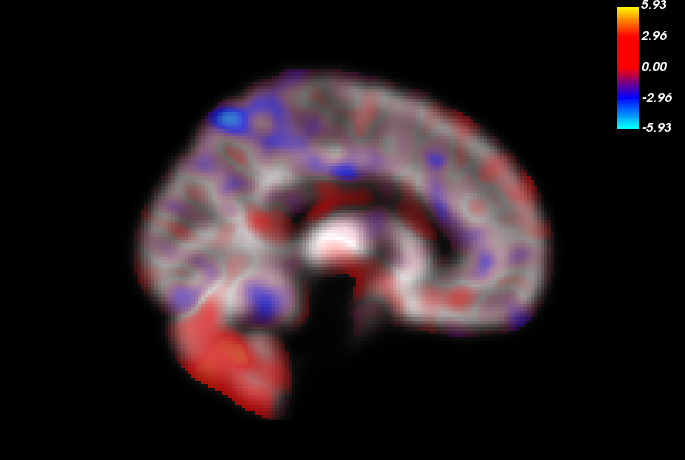

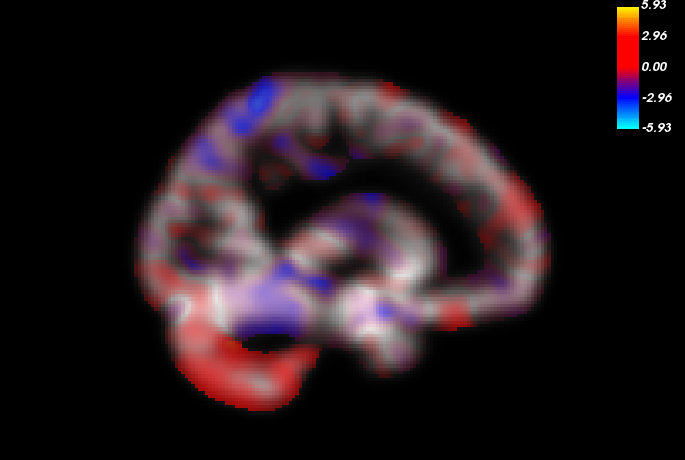

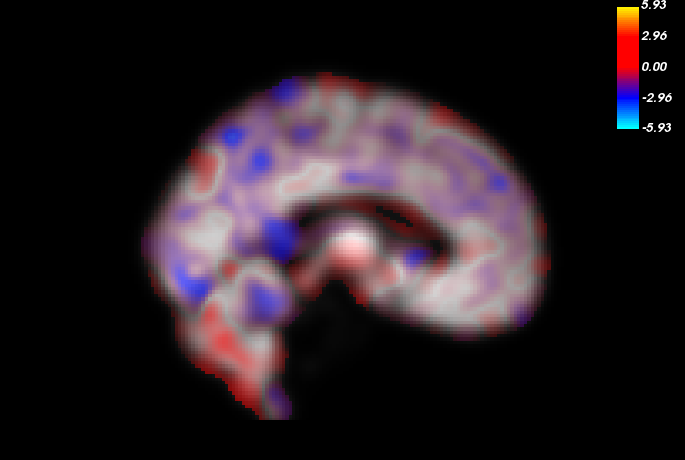

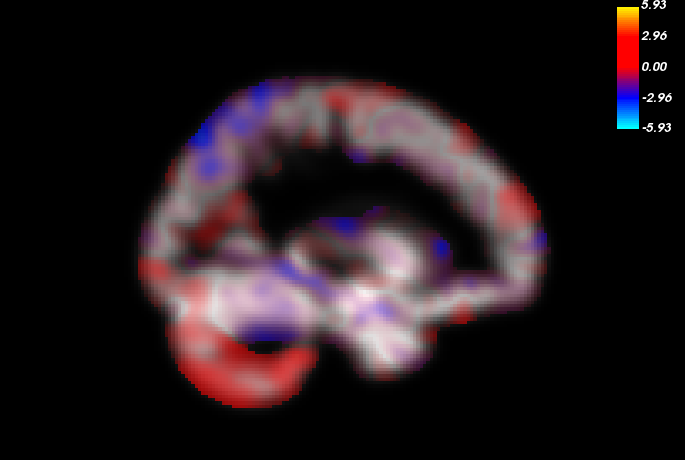

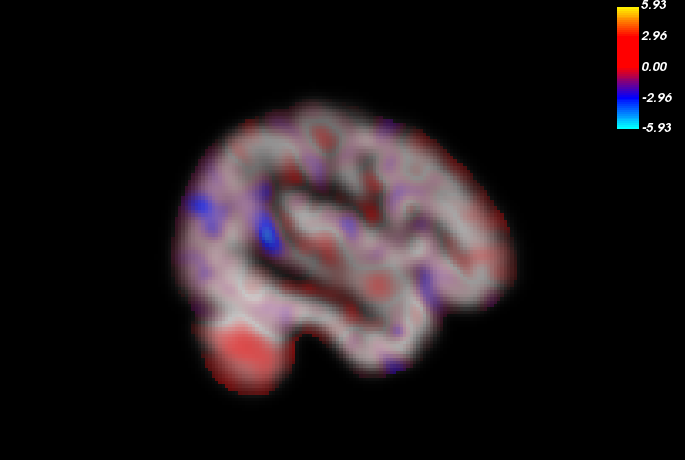

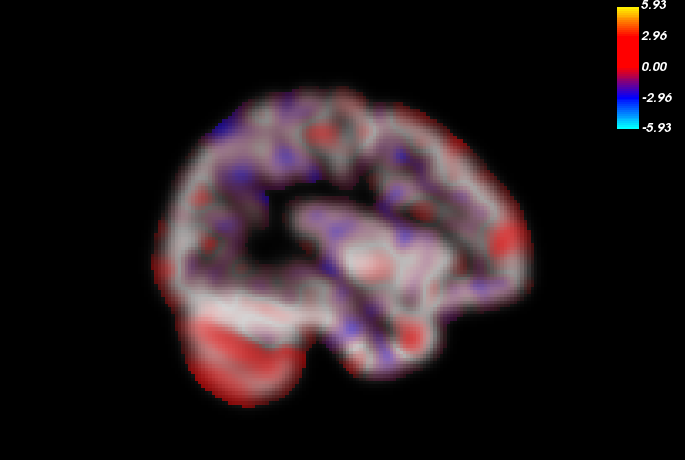

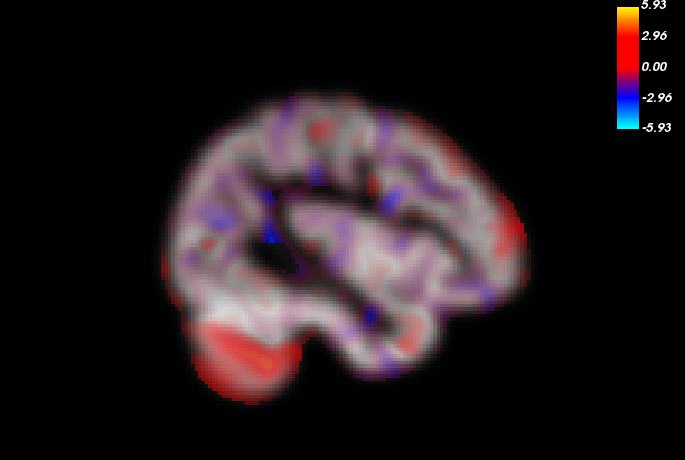

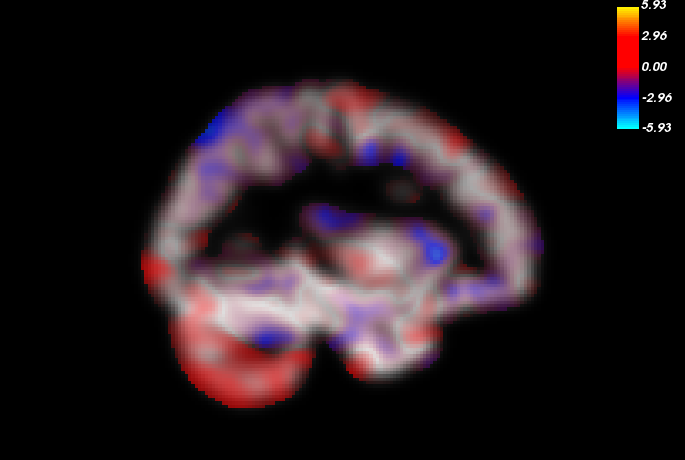

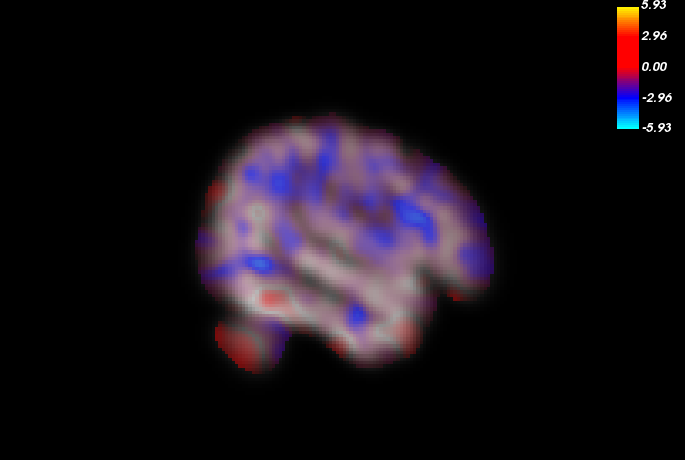


**Supplemental Figure 3. Gray Matter Volume.** Resulting t-stat maps representing the Group:Time interaction are overlaid on an average of all gray matter images. Warmer colors represent a greater t-stat value; cooler colors represent a greater negative t-stat. The waitlist control condition serves as the reference. The color gradient and legend are determined by the range of the data. Images were created using Freeview from Freesurfer version 7.4.1.


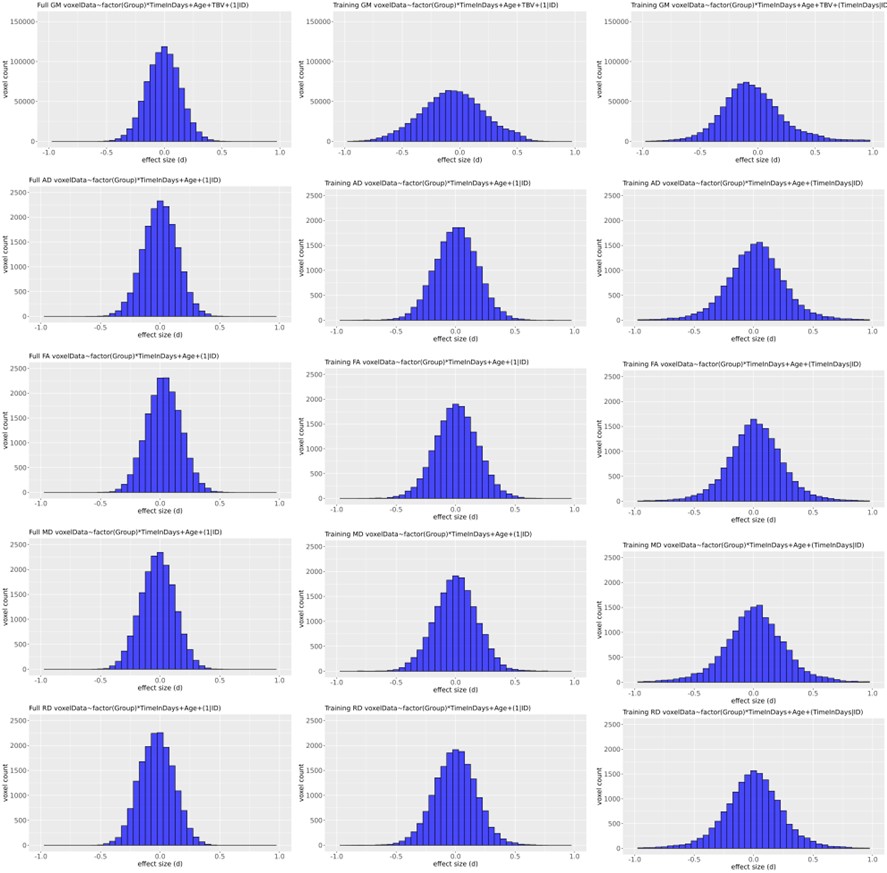
**Supplemental Figure 4.** Histograms of Cohen’s d calculated for the Group:Time interaction across all voxels for all the models run for the Full intervention (time points 1-6; left column) and the Training period only (including baseline, time points 1 – 4; right two columns). Top Row is Gray Matter, 2^nd^ row is Axial Diffusivity, 3^rd^ is Fractional Anisotropy, 4^th^ row is Medial Diffusivity and 5^th^ row is Radial Diffusivity. Note that the shape of the distribution is such that almost all is contained withing the medium to low effect size range.


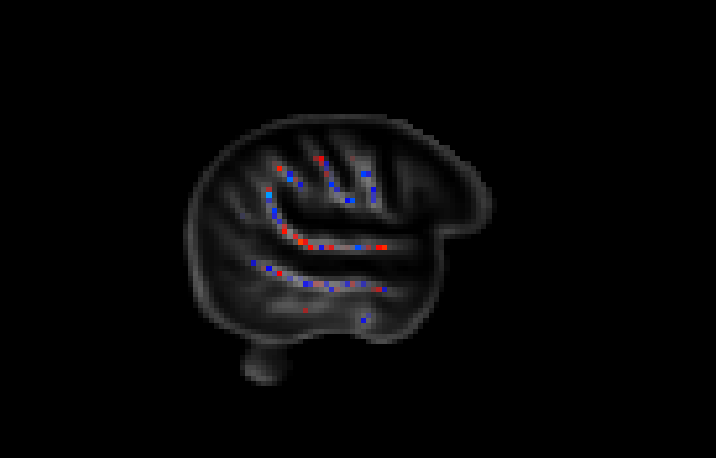

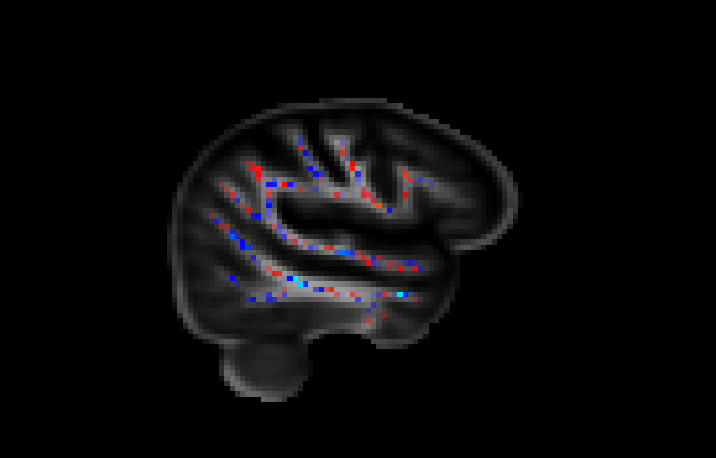

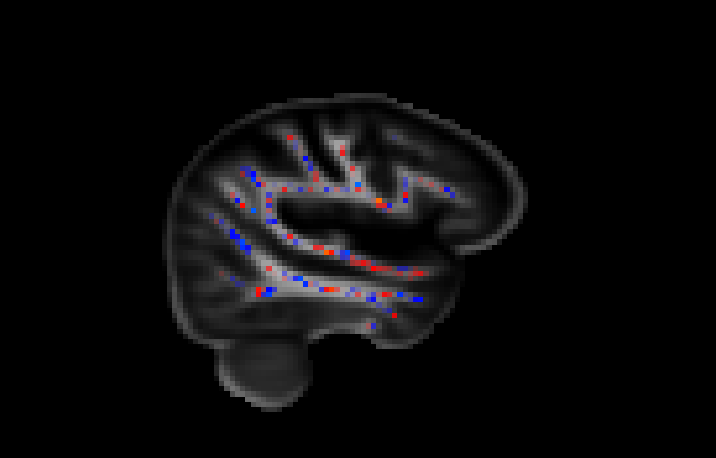

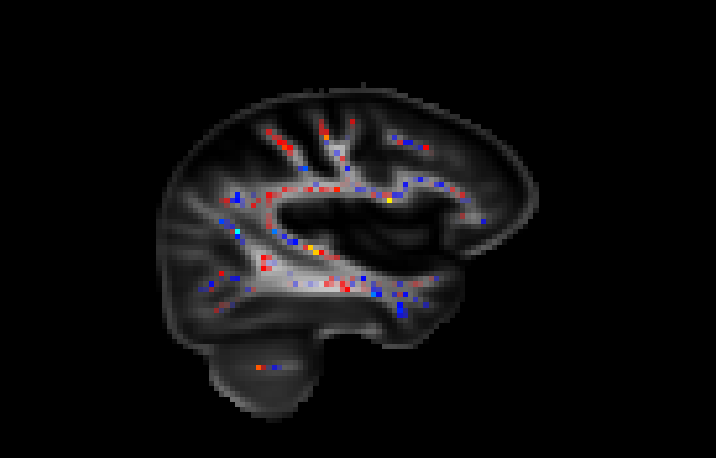

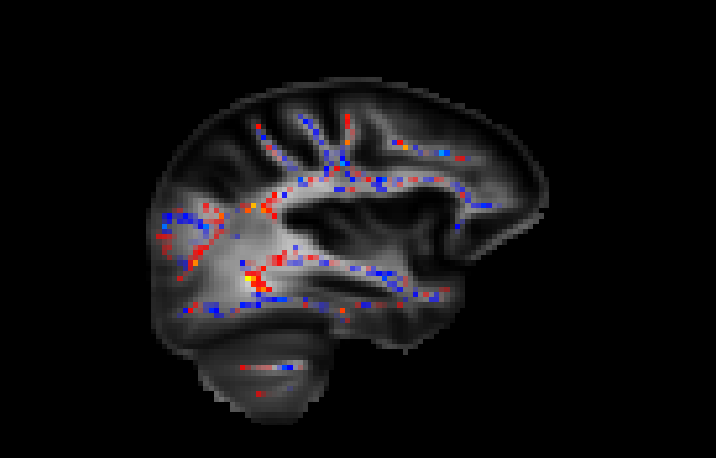

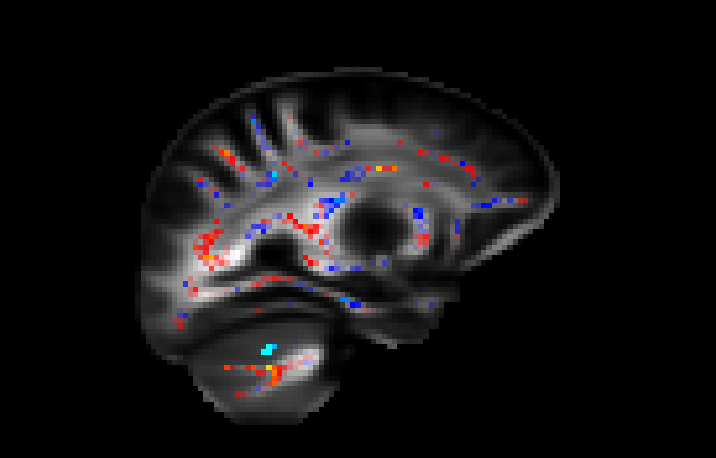

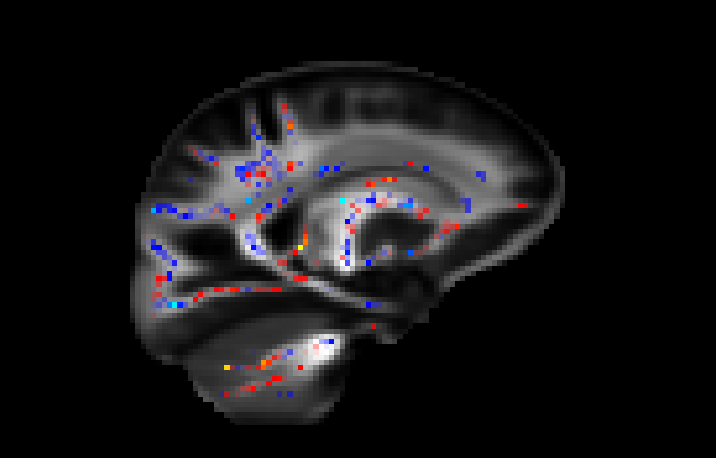

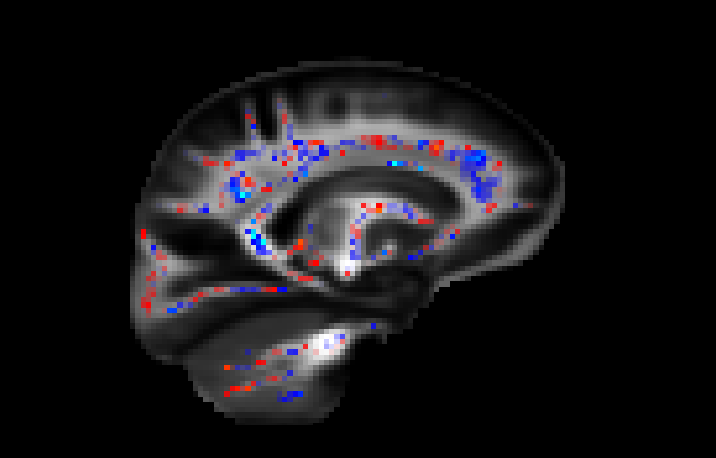

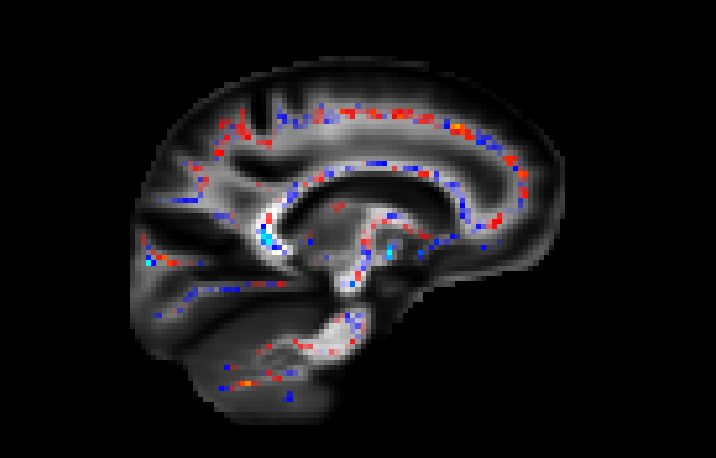

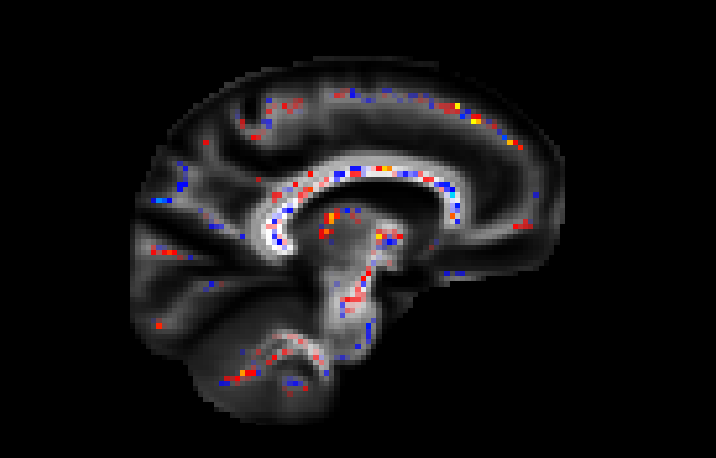

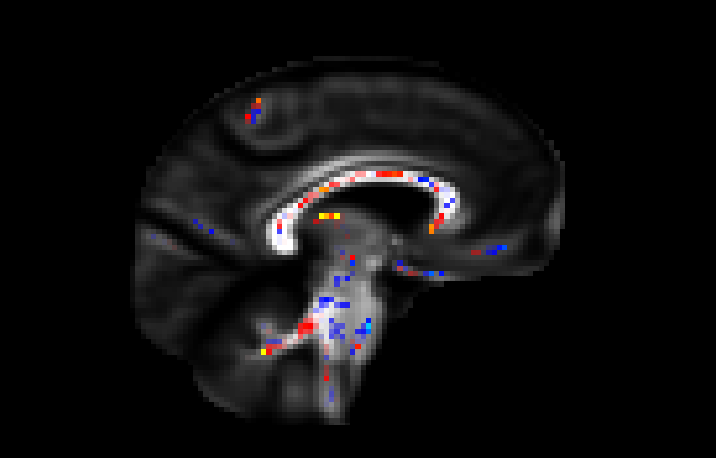

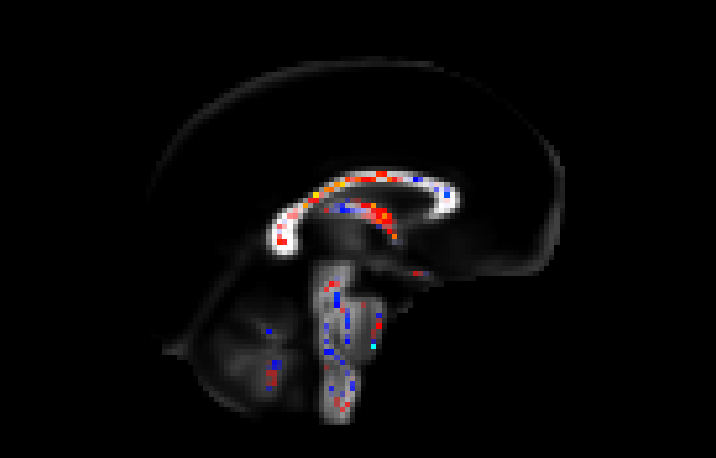

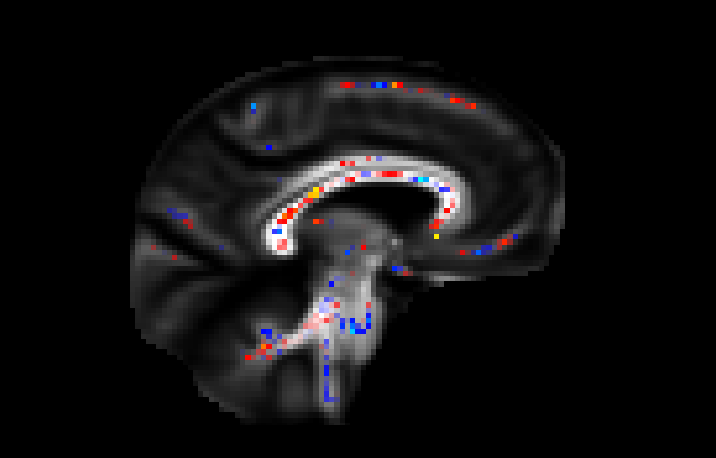

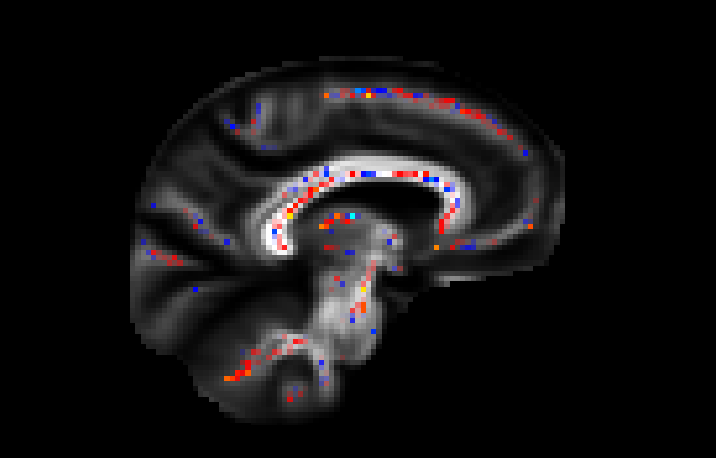

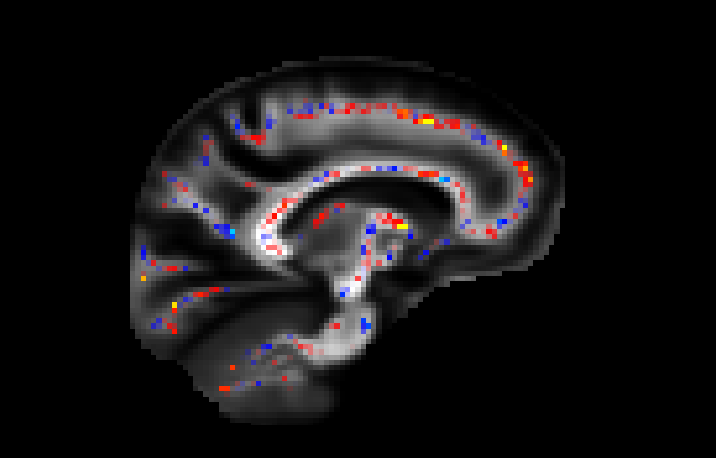

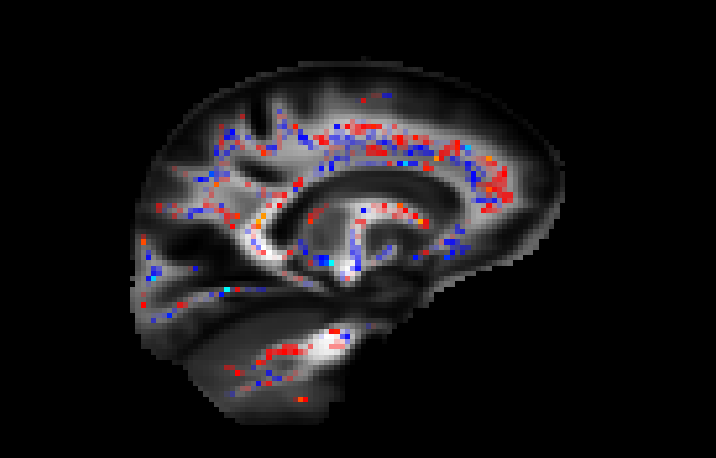

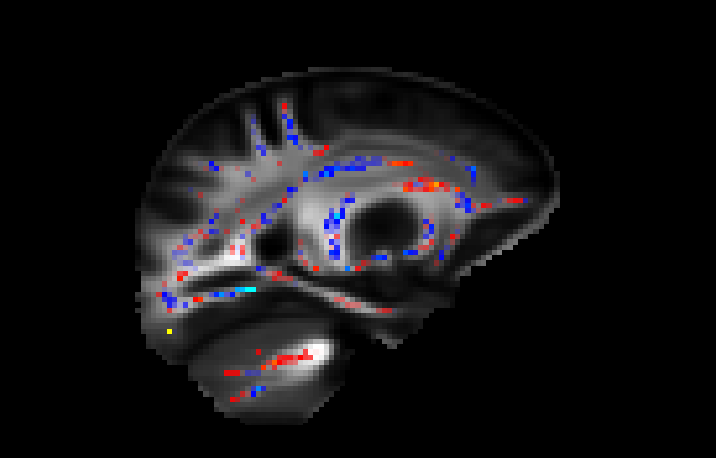

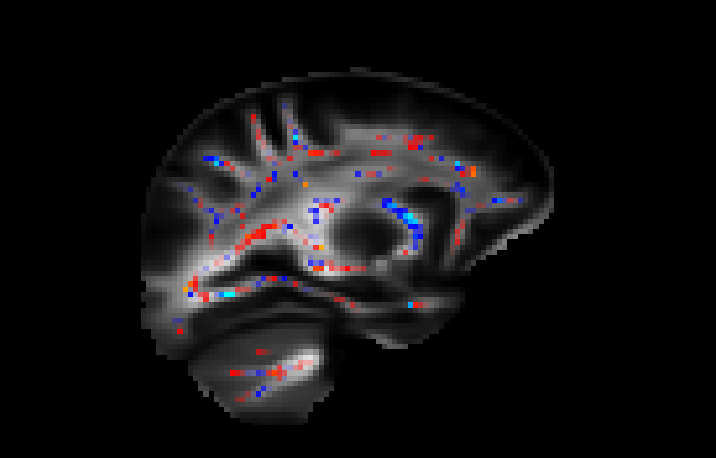

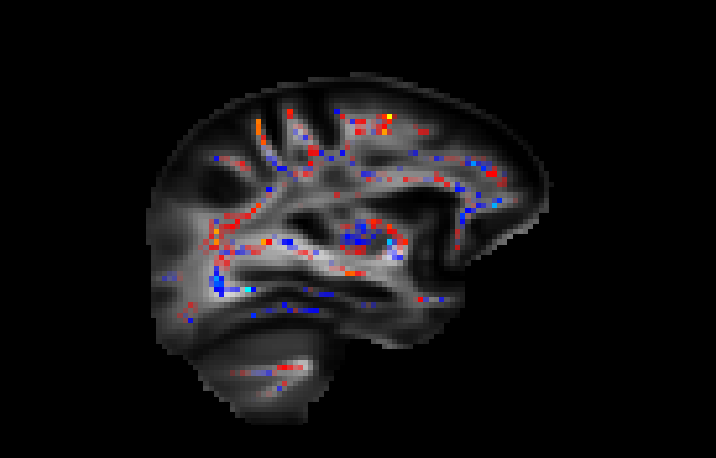

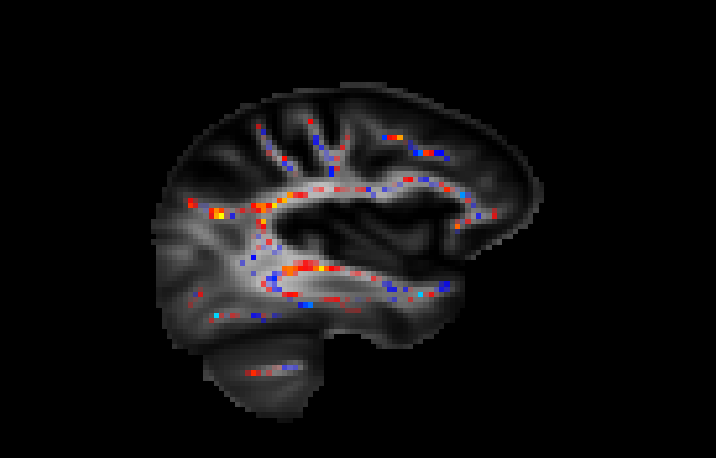

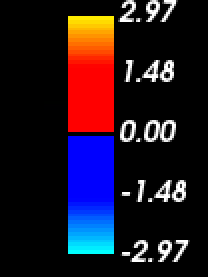


**Supplemental Figure 5. Fractional Anisotropy (FA).** Resulting t-stat maps representing the Group:Time interaction overlaid on the mean population image. Warmer colors represent a greater t-stat value; cooler colors represent a greater negative t-stat. The waitlist control condition serves as the reference. The color gradient and legend are determined by the range of the data. Images were created using Freeview from Freesurfer version 7.4.1.


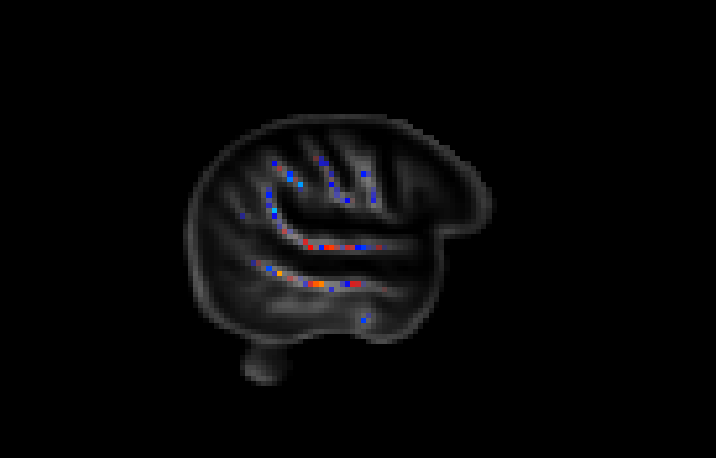

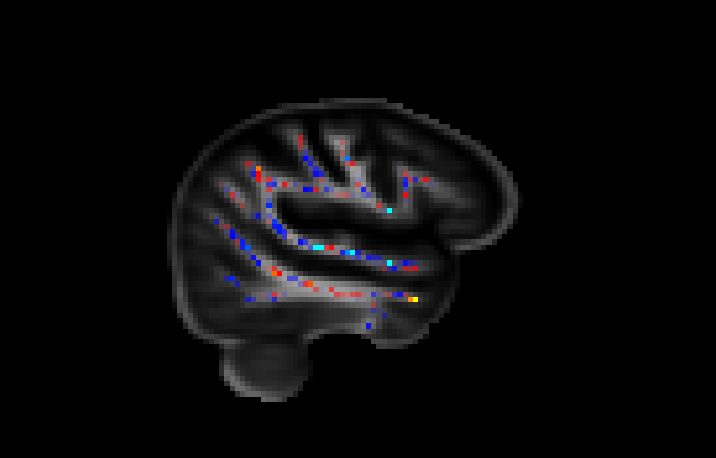

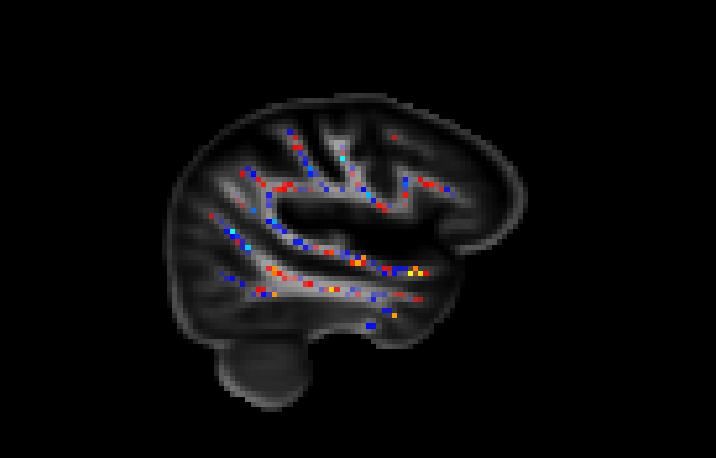

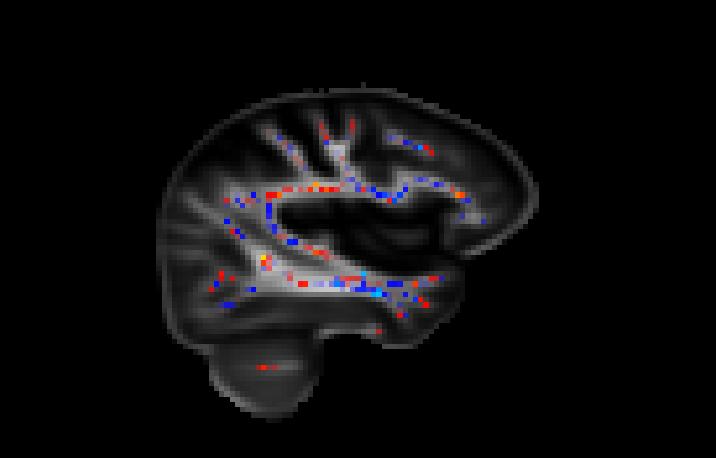

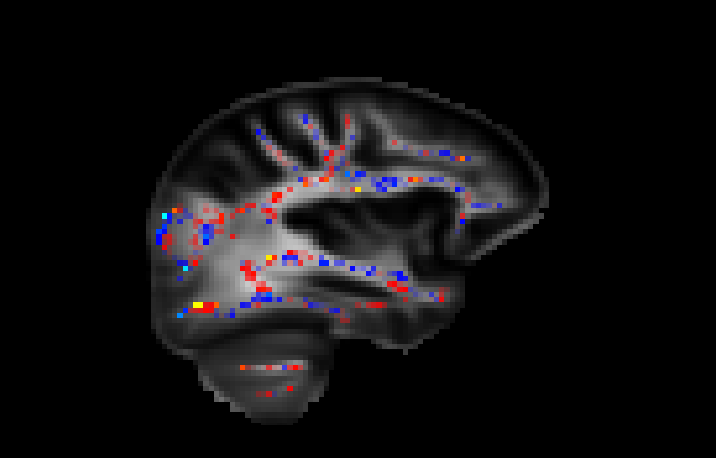

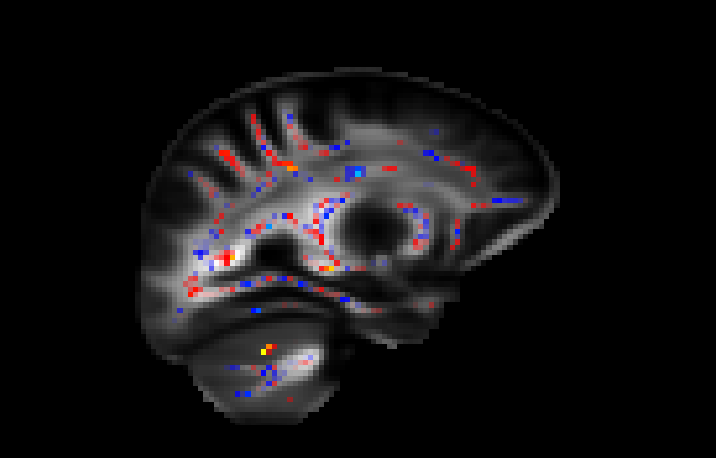

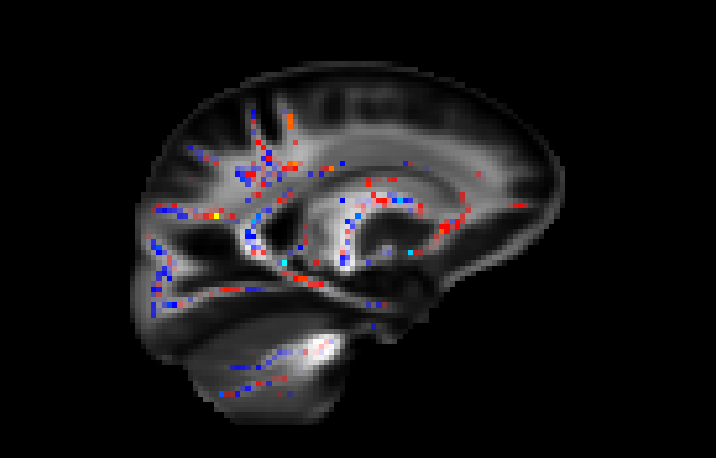

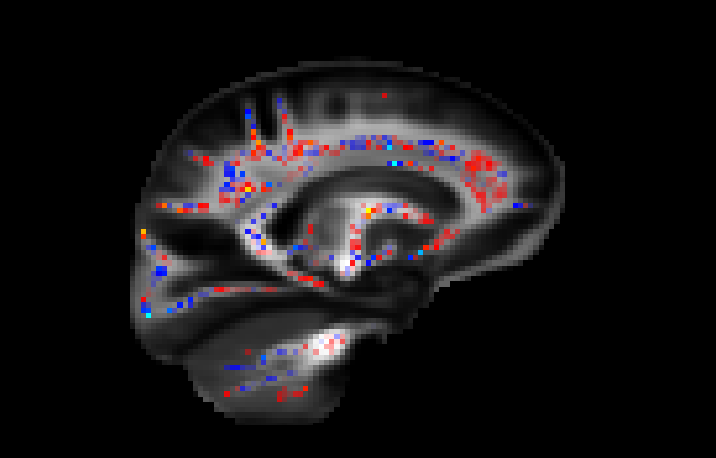

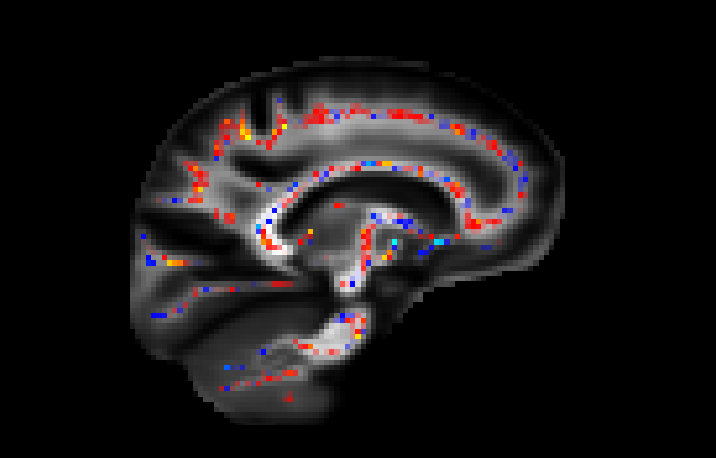

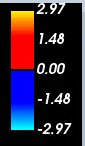

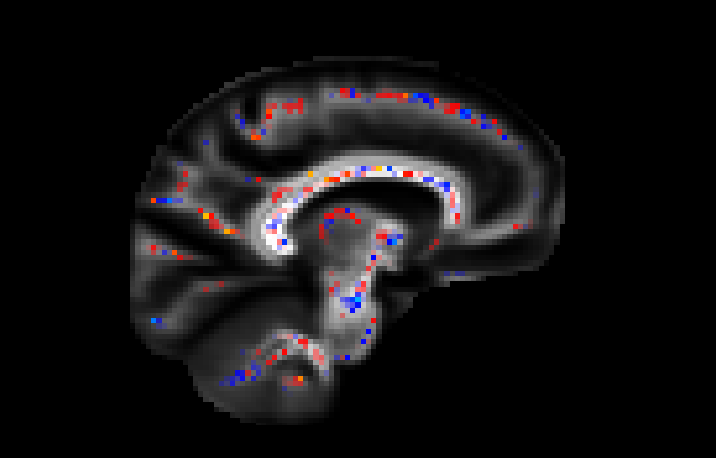

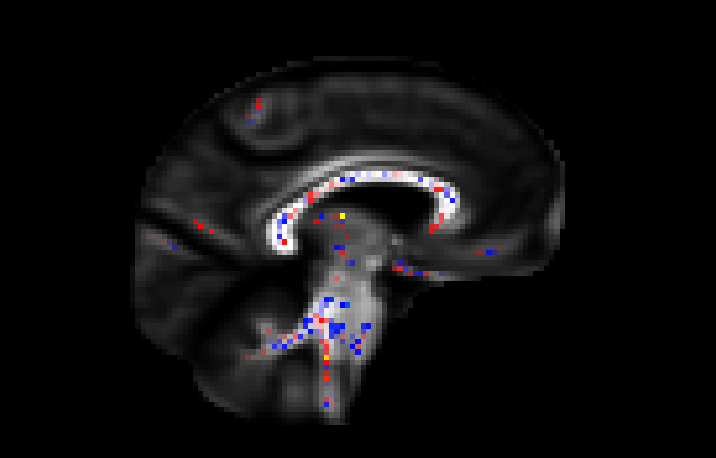

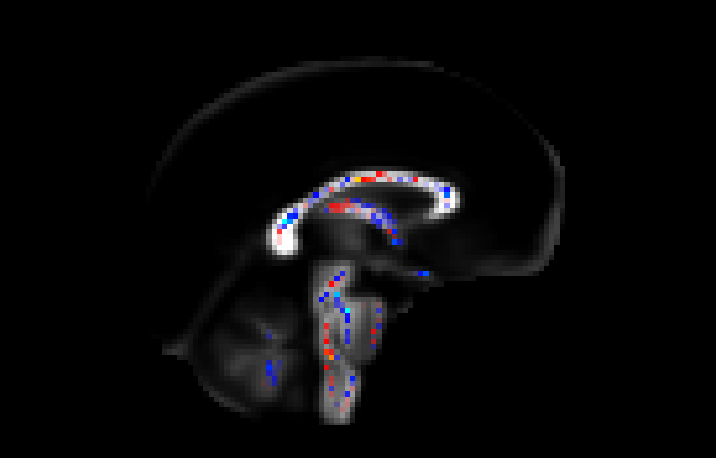

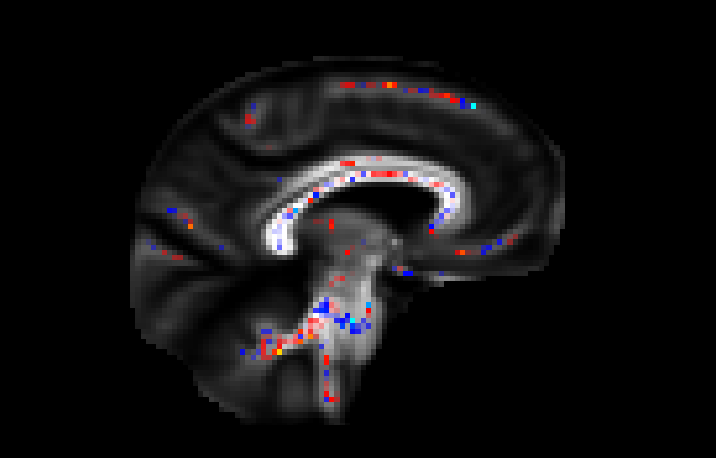

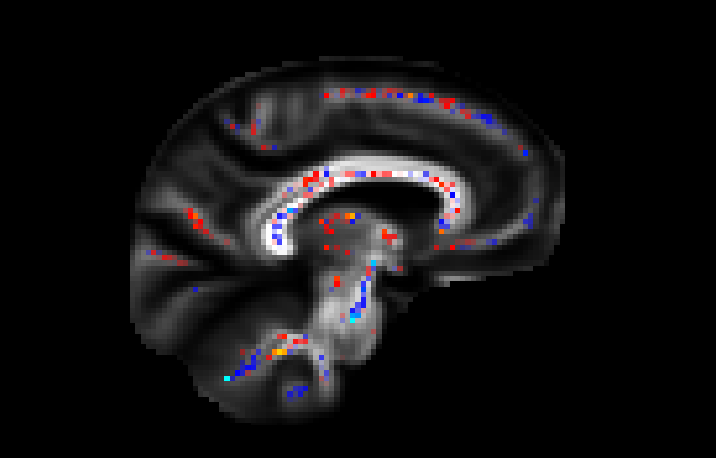

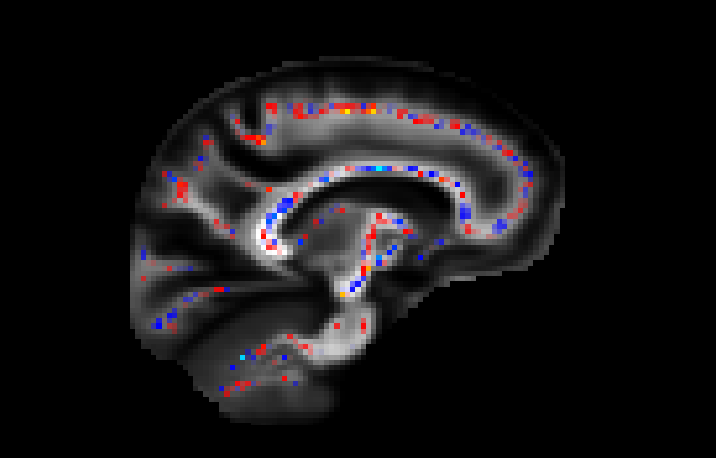

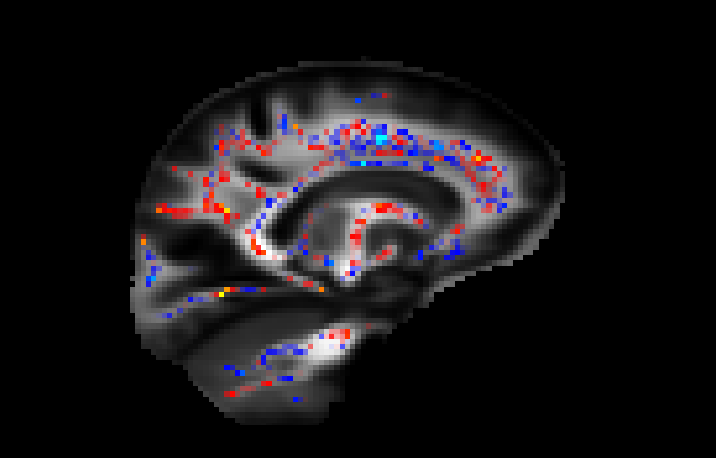

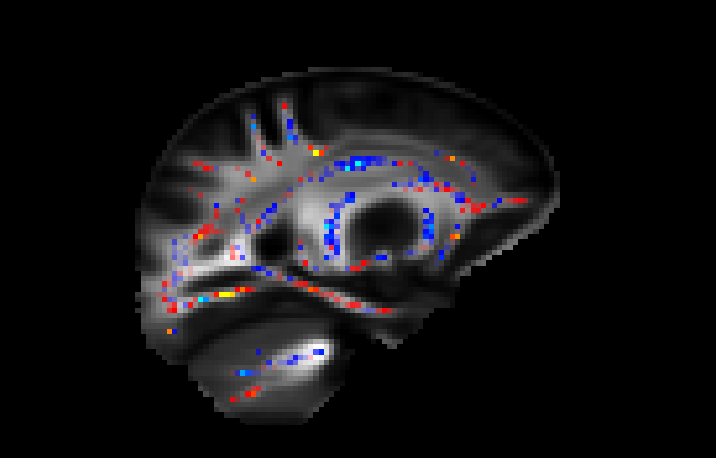

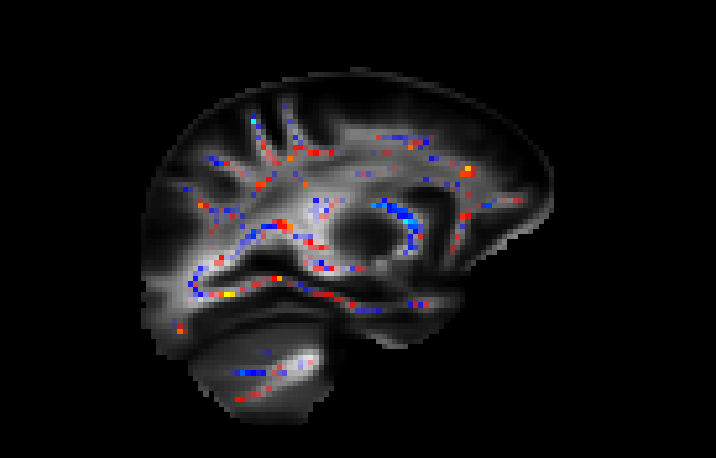

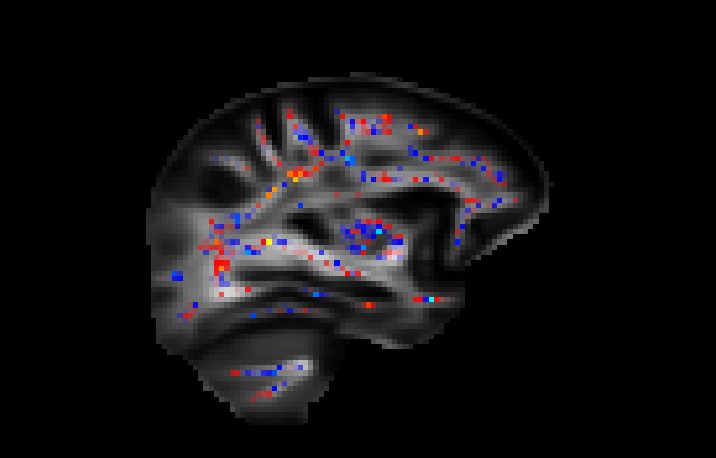

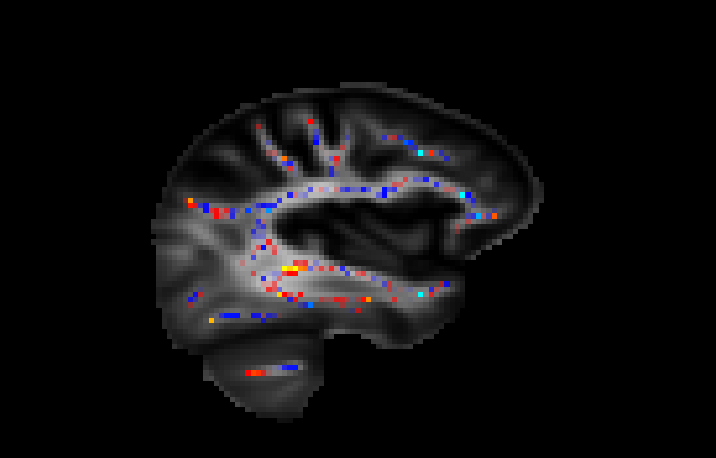


**Supplemental Figure 6. Axial Diffusivity (AD).** Resulting t-stat maps representing the Group:Time interaction overlaid on the mean population image. Warmer colors represent a greater t-stat value; cooler colors represent a greater negative t-stat. The waitlist control condition serves as the reference. The color gradient and legend are determined by the range of the data. Images were created using Freeview from Freesurfer version 7.4.1.


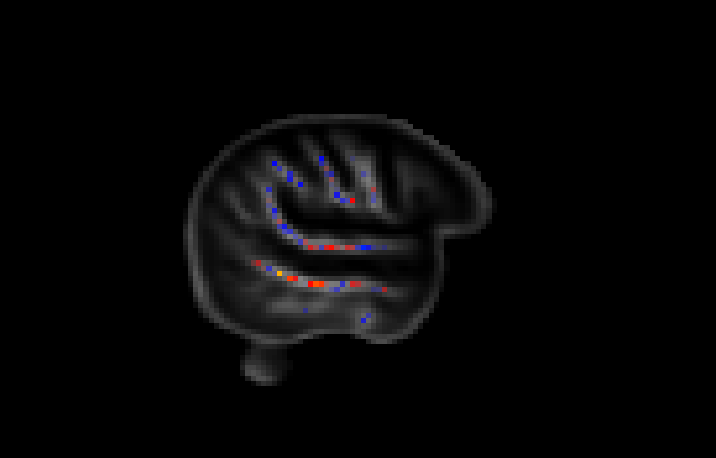

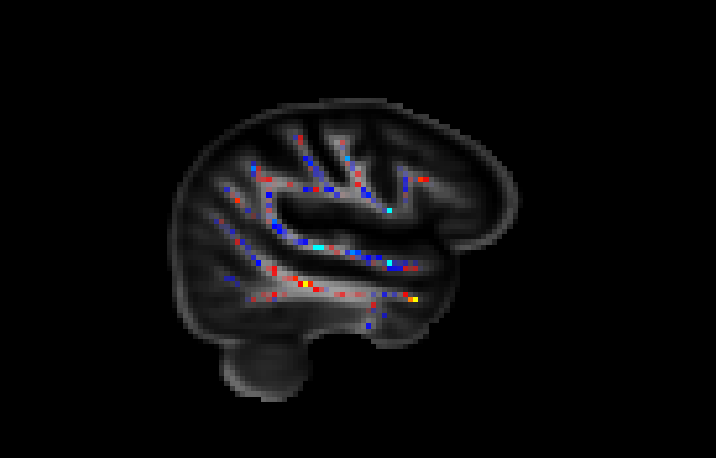

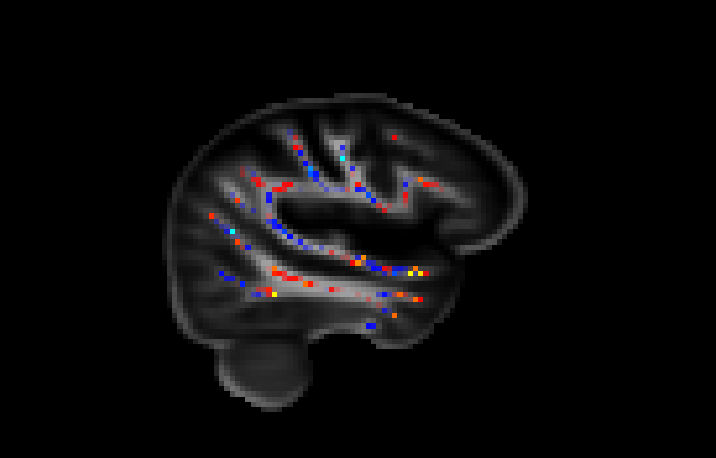

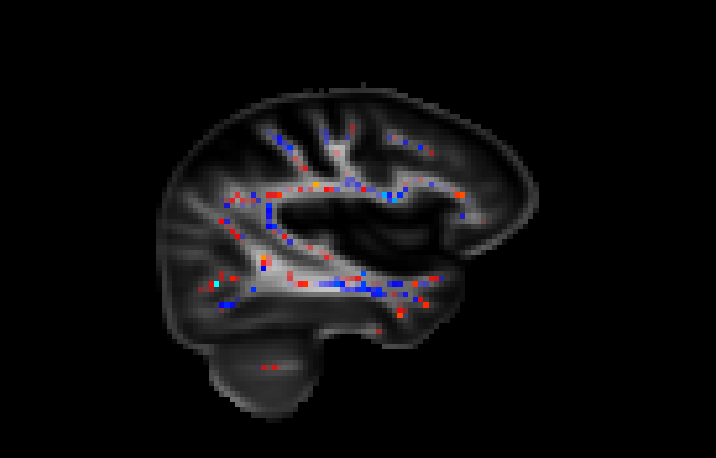

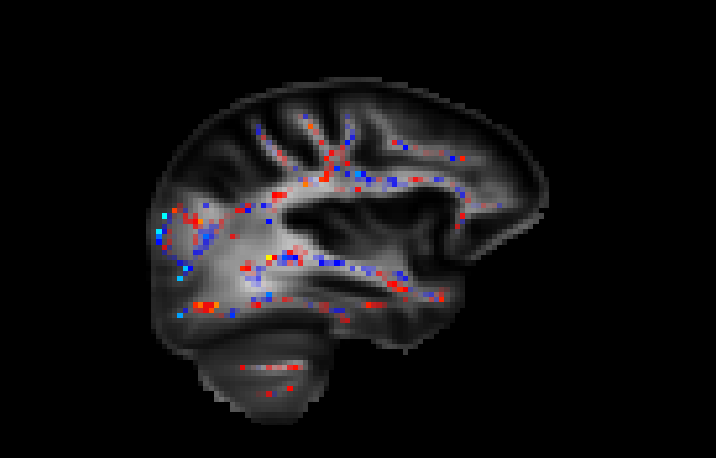

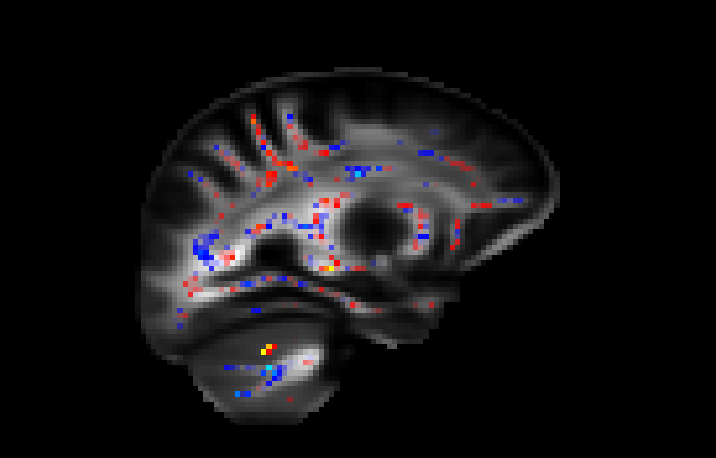

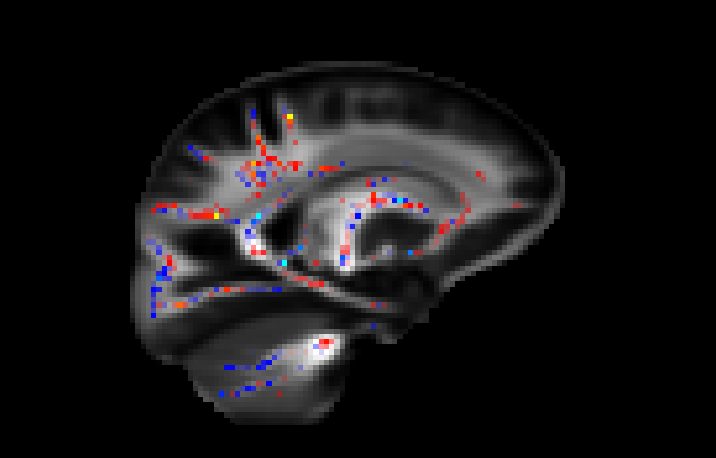

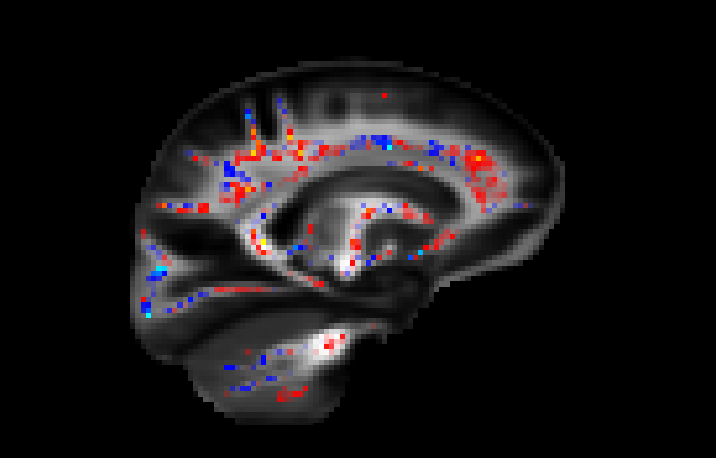

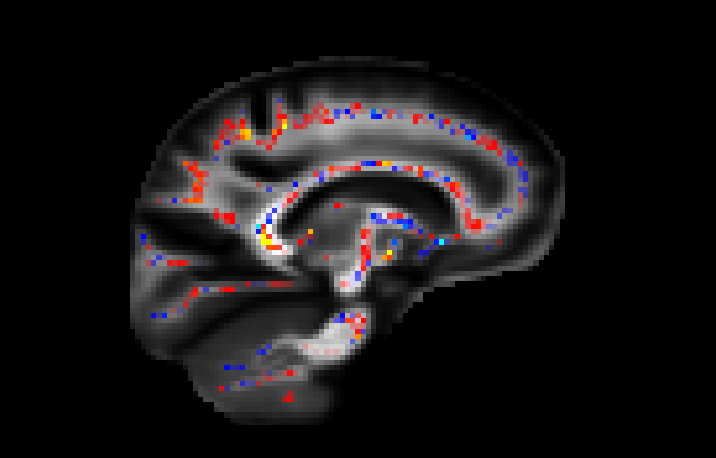

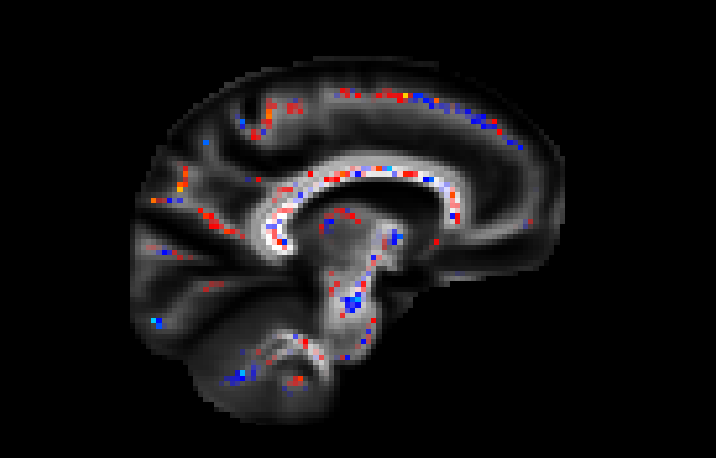

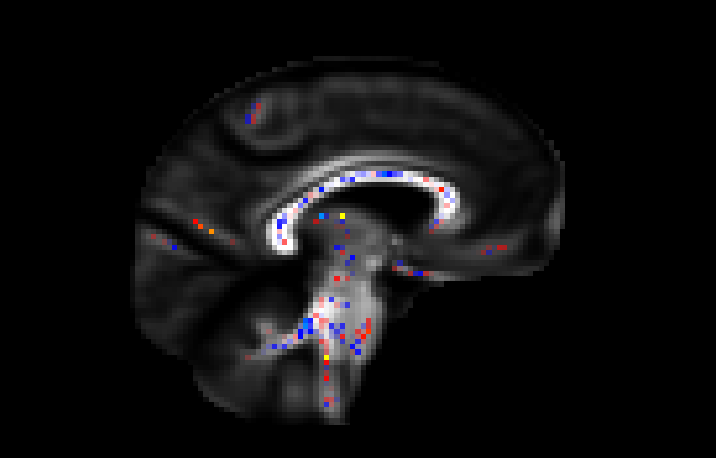

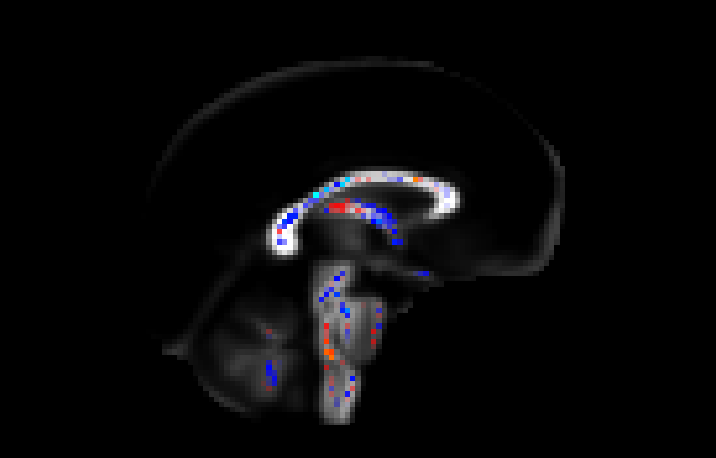

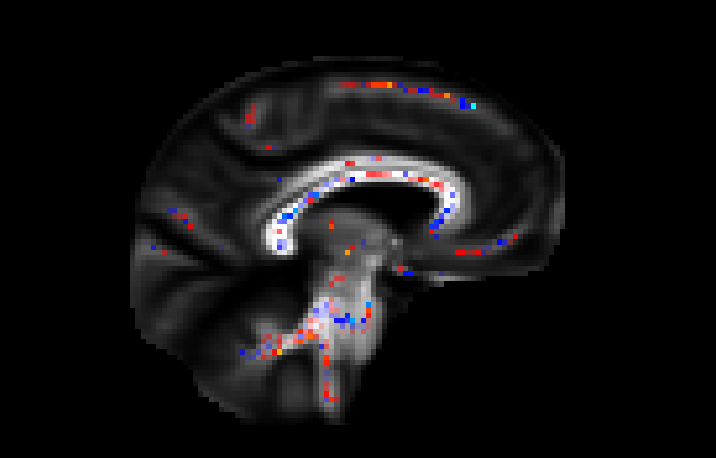

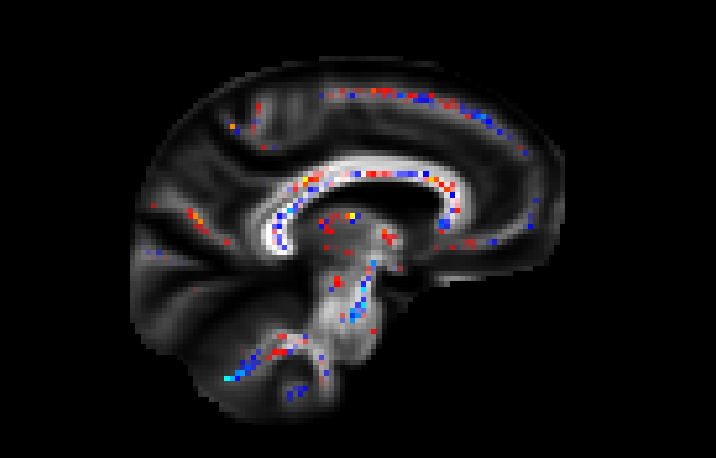

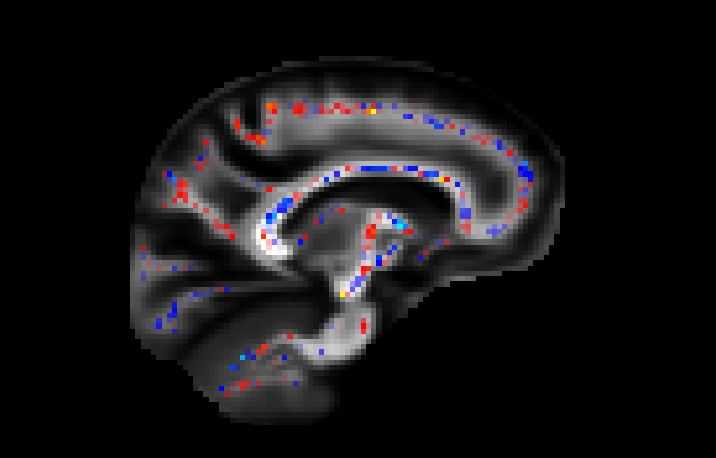

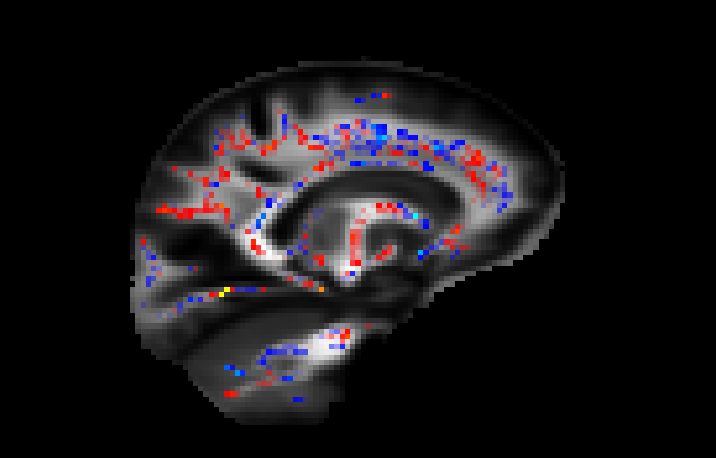

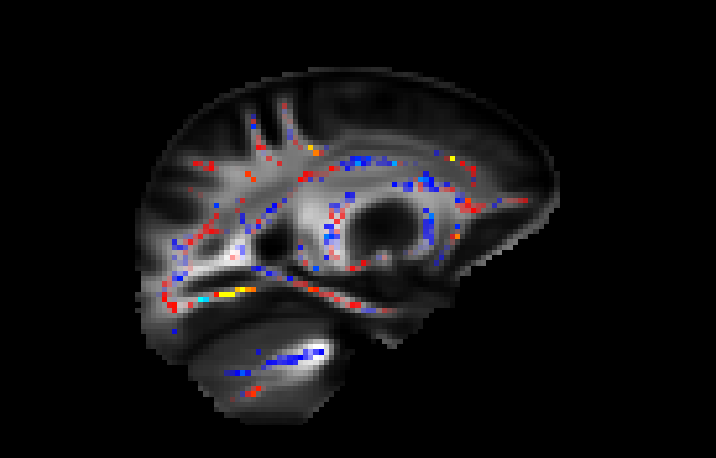

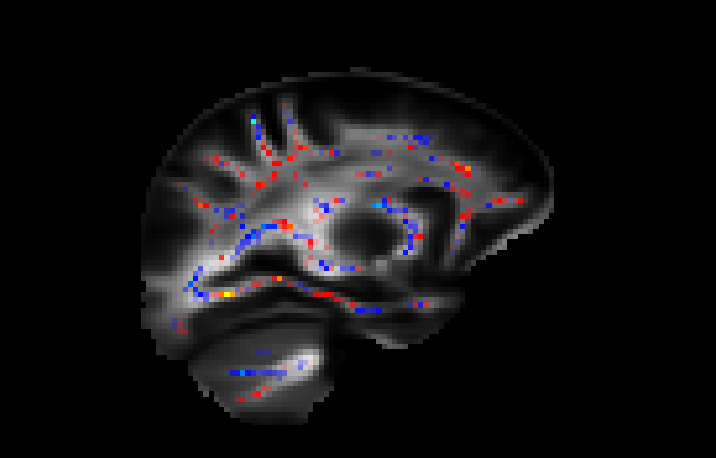

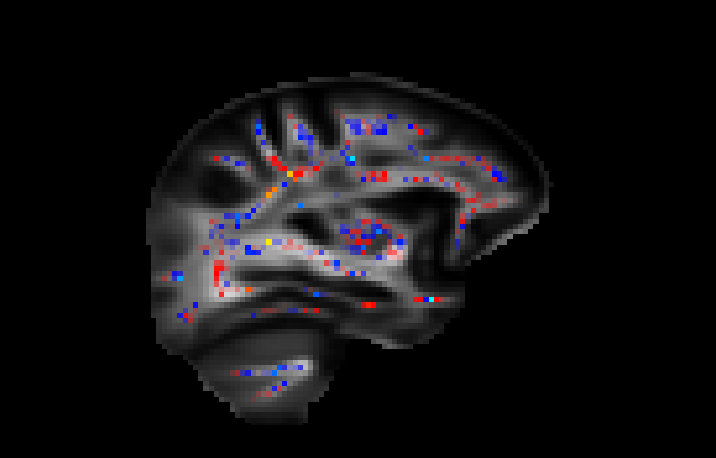

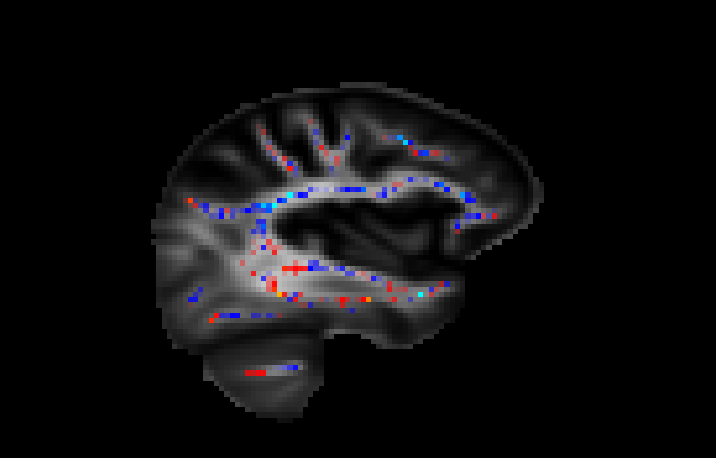

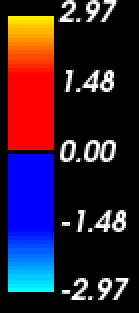


**Supplemental Figure 7. Mean Diffusivity (MD).** Resulting t-stat maps representing the Group:Time interaction overlaid on the mean population image. Warmer colors represent a greater t-stat value; cooler colors represent a greater negative t-stat. The waitlist control condition serves as the reference. The color gradient and legend are determined by the range of the data. Images were created using Freeview from Freesurfer version 7.4.1.


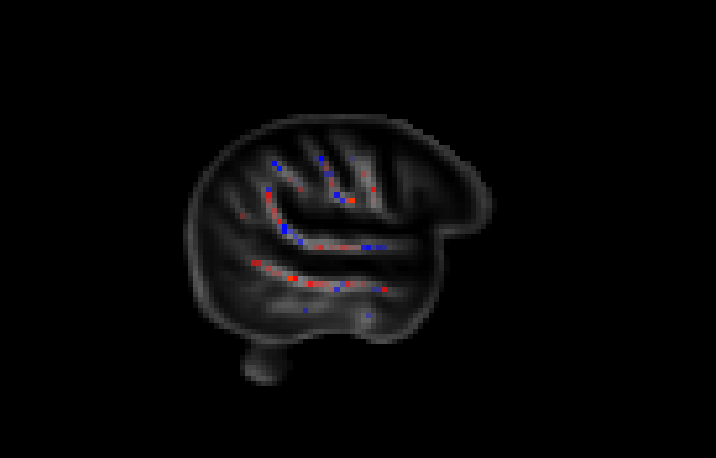

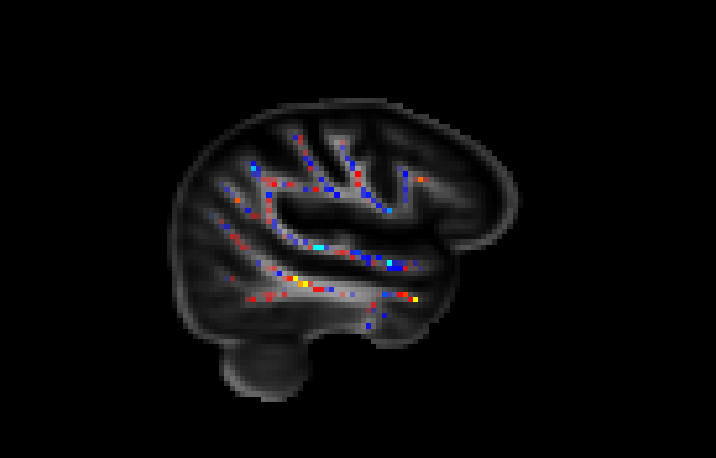

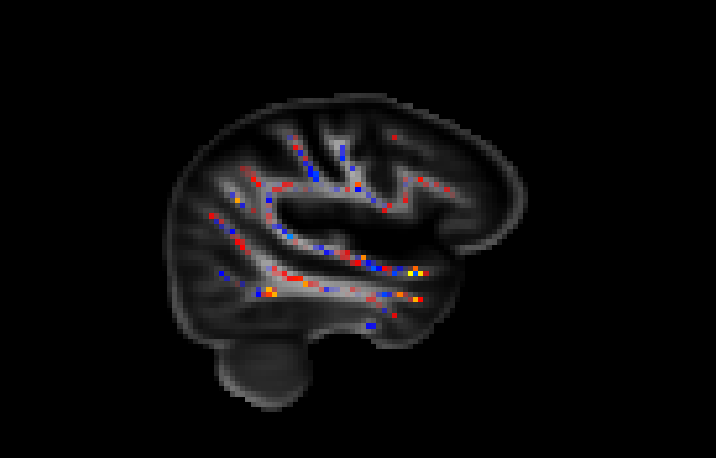

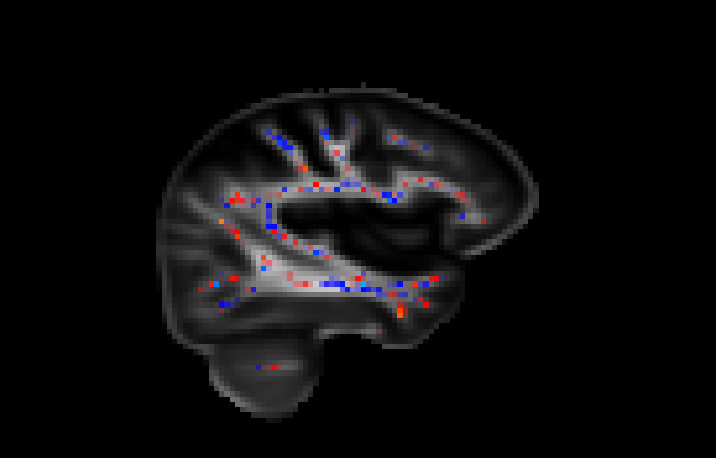

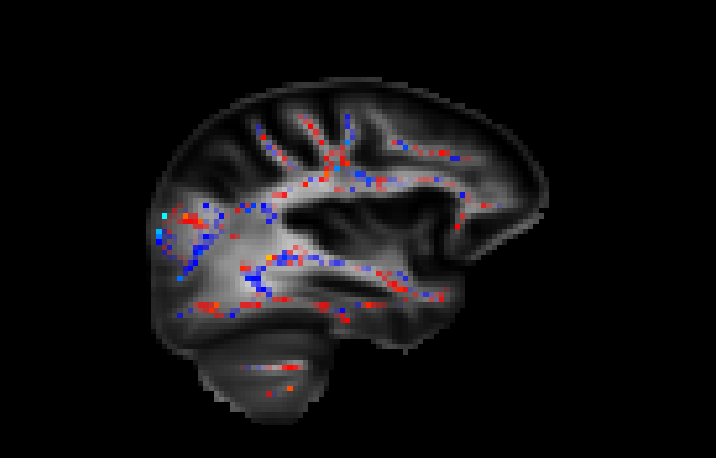

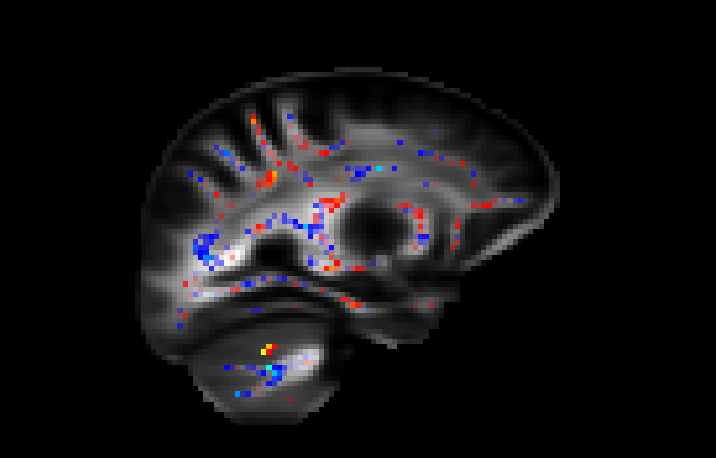

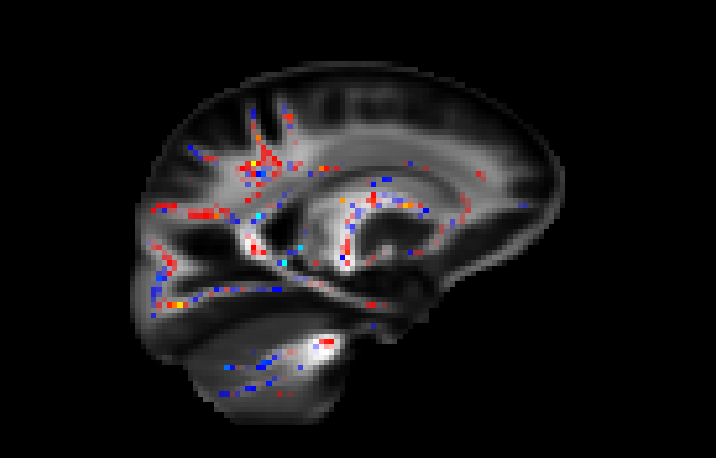

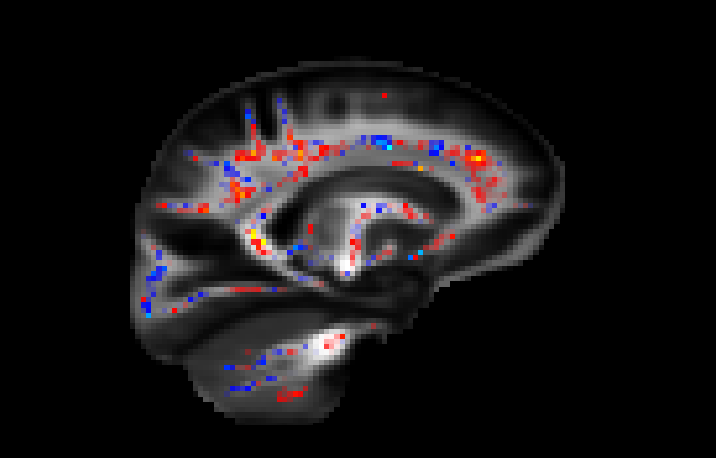

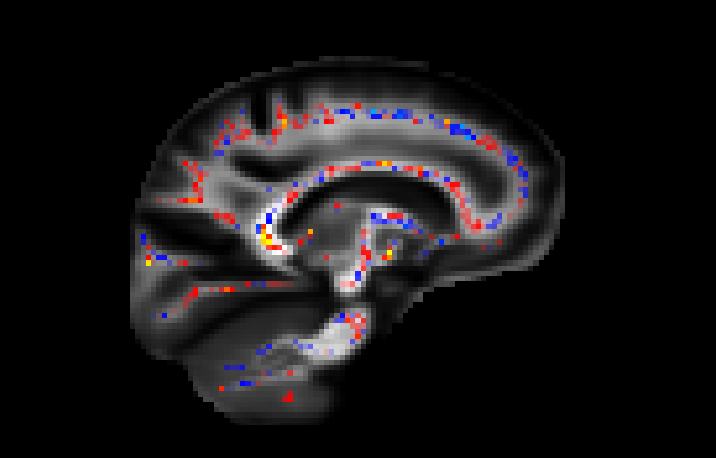

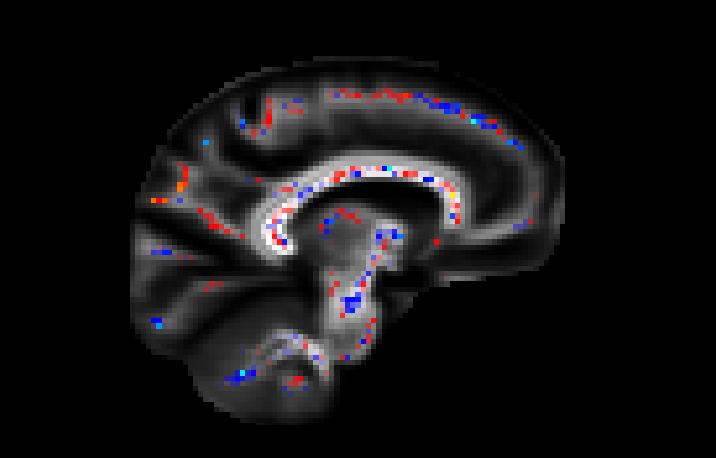

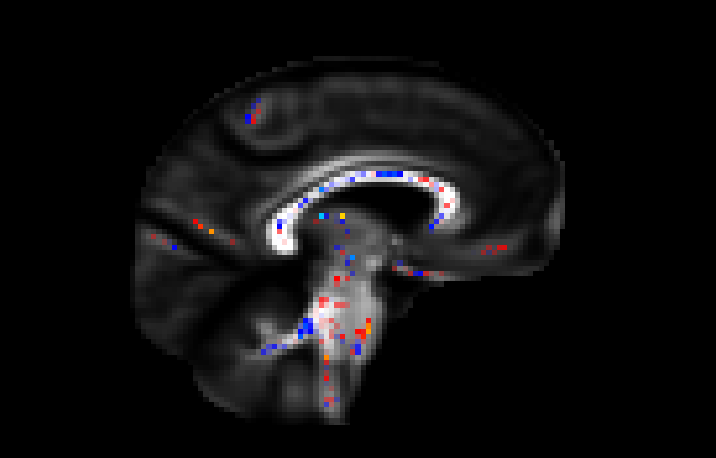

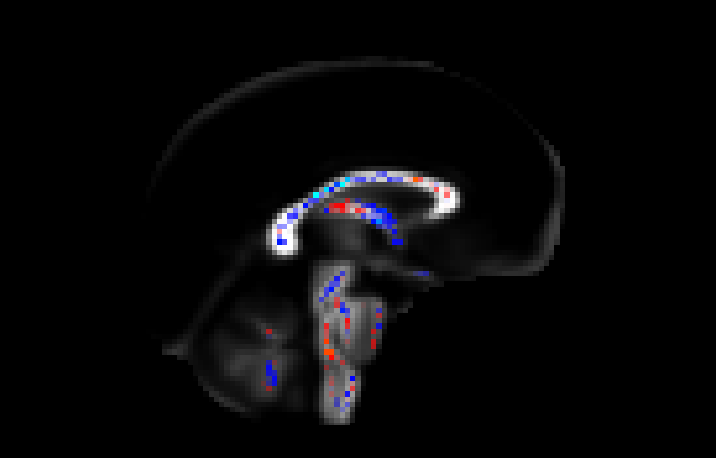

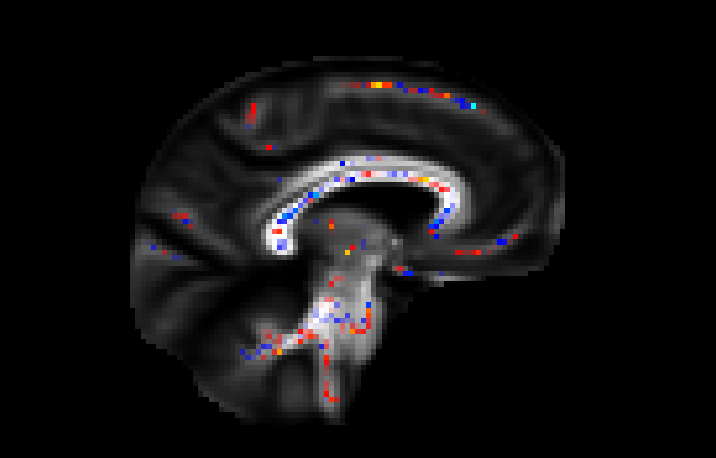

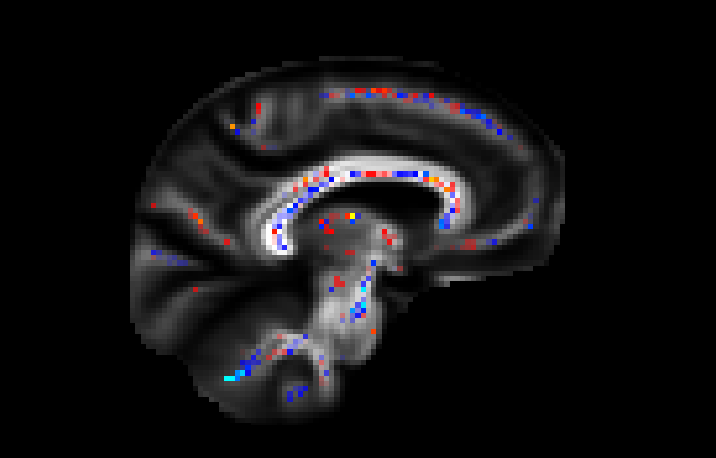


**Supplemental Figure 7. Mean Diffusivity (MD).** Resulting t-stat maps representing the Group:Time interaction overlaid on the mean population image. Warmer colors represent a greater t-stat value; cooler colors represent a greater negative t-stat. The waitlist control condition serves as the reference. The color gradient and legend are determined by the range of the data. Images were created using Freeview from Freesurfer version 7.4.1.
